# Features and predictive value of 6-min walk test outcomes in interstitial lung disease: an observation study using wearable monitors

**DOI:** 10.1136/bmjopen-2021-055077

**Published:** 2022-06-15

**Authors:** Jiaying Li, Xiaoyan Li, Miaozhen Deng, Xinyin Liang, Huiqun Wei, Xiaobing Wu

**Affiliations:** 1 School of Nursing, Li Ka Shing Faculty of Medicine, The University of Hong Kong, Hong Kong, Hong Kong; 2 Guangzhou Institute of Respiratory Health, First Affiliated Hospital of Guangzhou Medical College, Guangzhou, Guangdong, China; 3 Department of Internal Medicine, First Affiliated Hospital of Guangzhou Medical College, Guangzhou, Guangdong, China

**Keywords:** Interstitial lung disease, Rehabilitation medicine, RESPIRATORY MEDICINE (see Thoracic Medicine)

## Abstract

**Objectives:**

To describe 6-min walk test (6MWT) outcomes, and to investigate their correlations with cardiopulmonary and lung function among patients with interstitial lung disease (ILD) which was not limited to idiopathic pulmonary fibrosis.

**Methods:**

We collected patients’ demographic data and obtained minute-by-minute 6MWT outcomes. Modified Borg scale was employed to assess patients’ dyspnoea, whereas New York Heart Association (NYHA) classification and pulmonary function test were used to evaluate patients’ cardiopulmonary functions.

**Results:**

Heart rate (HR) exhibited a continuous upward trend, while SpO_2_ exhibited an overall downward with a slight increase at the fifth minute. The SpO_2_ nadir for 70 patients (9.3%) was lower than 80%. Further, the SpO_2_ nadir for 78.27% of the participants appeared at the end of the fourth minute. The 6-min walk distance (6MWD) had the strongest correlation with NYHA classification (*r=*0.82, p*<*0.01). The ratio of 6MWD to predicted 6MWD was most correlated to forced expiratory volume in the first second (*r=*0.30, p*<*0.01) and forced vital capacity (*r=*0.30, p*<*0.01). SpO_2_ at 3 min had the strongest correlation to patients’ diffusing capacity of the lungs for carbon monoxide (*r=*0.41, p*<*0.01). We found significant differences in 6MWD (*F*=2.44, p=0.033), SpO_2_ change (*F*=2.58, p=0.025), HR at 0 min (*F*=2.87, p=0.014), HR at end of 6 min (*F*=2.58, p=0.025) and HR zenith (*F*=2.64, p=0.022) between the subtypes of ILD.

**Conclusion:**

This observation provided an important evidence regarding oxygen titration. It is better to maintain SpO_2_ above 88% for 4 min instead of 3 min. SpO_2_ at the third minute was the most valuable predictor of patients’ lung function. 6MWD and SpO_2_ changes were more discriminative in subtypes.

**Strengths and limitations of this study**

- The study included a large sample size of patients with interstitial lung disease (ILD) not limited to idiopathic pulmonary fibrosis.
- This was the first study to compare 6-min walk test (6MWT) outcomes between multiple subtypes of ILD.
- This study described the tendency of heart rate and SpO_2_ minute-by-minute during 6MWT.
- Lack of follow-up hindered the prediction of patients’ long-term clinical outcomes using 6MWT.

## Background

Interstitial lung disease (ILD) is a group of more than 200 kinds of diseases characterised by pulmonary inflammation, accompanied with or without fibrosis.^1 2^ Patients diagnosed with ILD mostly have dyspnoea and decreased tolerance to exercise.^3^ The 6-min walk test (6MWT) is widely used to assess patients’ performance ability with different cardiopulmonary-related diseases, which provides essential outcomes that cannot be obtained otherwise by standardised pulmonary function testing.^4^

Until, most 6MWT-related studies have focused on idiopathic pulmonary fibrosis (IPF), which is the most common type of ILD. Previous studies have investigated the outcomes of 6MWT, most of which centred on the 6-min walk distance (6MWD) and percutaneous oxygen saturation (SpO_2_). However, 6MWT is ideal in predicting patients’ clinical outcomes. Previous studies found that 6MWD and oxygen desaturation are associated with mortality in patients with IPF.^5 6^ According to a previous study, 6MWD was an independent positive factor for the physical activity of patients with IPF.^7^ In addition, 6MWD had a positive association with the subjective health-related quality of life (HRQL) and objective lung function index,^8 9^ which included the predicted percentage of forced vital capacity (FVC) and predicted lung diffusing capacity for carbon monoxide (DLco)^5 8 9^ as well as forced expiratory volume in the first second (FEV_1)_,^10^ which is a negative predictor for dyspnoea.^8 9 11 12^ Furthermore, the occurrence of desaturation and changes in SpO_2_ during the test were indicators of patients’ mortality with IPF.^13 14^

6MWT has multiple associated outcomes that are not restricted to 6MWD and SpO_2._ It comprises the distance walked, heart rate (HR), blood pressure, SpO_2_ and dyspnoea, as assessed by the Borg scale.^15^ Although the predictive value of oxygen desaturation for the clinical outcomes of the patients has been confirmed,^5 6^ the most predictive time point of this outcome within the 6 min remains unclear. In addition, the effect of different subtypes of ILD on 6MWT outcomes has not been evaluated yet. Several studies highlighted the importance of finding the most prognostic outcome of 6MWT,^6 16^ and measuring SpO_2_ for the entire 6 min duration of 6MWT is recommended by the 2014 technical standards of European Respiratory Society and American Thoracic Society.^17^ In the current study, wearable monitors were used to obtain the precise minute-by-minute data of 6MWT, which facilitated the descriptions and comparisons in detail. The comparison between subgroups of ILD will provide new insights into the distinguishing value of 6MWT outcomes.

Hence, to provide detailed features, the predictive value of 6MWT for cardiopulmonary functions, and its distinguishing value for the subtypes of ILD in the current study, we aimed to: (1) describe the detailed outcomes of 6MWT outcomes, including the HR, SpO_2_, blood pressure, Borg score and walking distance. (2) Identify the correlations between 6MWT outcomes and patients’ cardiopulmonary functions. (3) Investigate the effect of the differences between subclassifications of ILD on 6MWT outcomes.

## Methods

### Design

This was an observational study using a wearable monitor.

### Patients

All the patients were recruited from July 2019 to August 2020 at the Guangzhou Respiratory Health Institute, the biggest respiratory centre in China. We identified eligible participants based on the following inclusion and exclusion criteria—we included patients who were diagnosed with ILD, or whose condition was feasible to conduct 6MWT. The expert pulmonologist established the diagnosis based on patients’ symptoms, the radiologist’s opinion from the imaging tests, blood tests results, lung function tests, bronchoscopy and biopsy. We excluded patients who had walking limitations, including joint restrictions or other critical diseases and those who experienced myocardial infarction in the previous 5 days, unstable angina, syncope, symptomatic arrhythmia, severe aortic stenosis or decompensated heart failure due to another unstable medical issues.^17^ After the initial screening, we obtained informed consent from eligible participants before including them in the study.

### Measurements

#### Demographics questionnaire

The self-designed demographic questionnaire included questions about the age, height, weight, body mass index and sex of the participants.

#### NYHA functional classification

The New York Heart Association (NYHA) classification was considered as a critical criterion for a comprehensive cardiac diagnosis.^18^ It classifies patients into four categories, based on their limitations during physical activity, which ranges from no symptoms with ordinary physical activity (class I) to symptoms at rest and increased discomfort with any physical activity (class IV).^19^

#### Borg scale

The Borg Rating of Perceived Exertion scale was developed by Borg,^20^ which is widely used to measure patients’ effort and exertion, breathlessness and fatigue during physical work. A higher score indicated a more severe level of exertion.^21^

#### Outcomes of 6MWT

According to Enright’s recommendations,^22^ the primary outcome in our study was 6MWD. We calculated the predicted 6MWD based on equations developed by Enright and Sherrill.^23^ For men, the predicted 6MWD=(7.57 * height_cm_)−(5.02 * age)−(1.76 * weight_kg_)−309 m. For women, the predicted 6MWD=(2.11 * height_cm_)−(2.29 * age)−(5.78 * weight_kg_)+667 m. Secondary outcomes include fatigue and dyspnoea, arterial oxygen saturation, HR and blood pressure. We measured fatigue and dyspnoea by modified Borg scale before and after 6MWT, and used wearable monitors to record patients’ arterial oxygen saturation and HR during 6MWT. We also recorded the patients’ blood pressure before and after the test, and calculate the mean arterial pressure. In this study, 6MWT was conducted without oxygen therapy support. Most of participants had received a 6MWT at the outpatient clinic before their hospital admission. In this case, the learning effect that improves the distance of second walk will be weak.^24^ Therefore, we conducted one 6MWT for each patient.

#### Pulmonary function test

Restriction of lung volumes and dysfunction of diffusion are the main functional respiratory abnormalities. An increased FEV_1_/FVC ratio, accompanied by a low total lung capacity, indicates restriction of lung volumes. Previous studies have proven that reduction in FVCand DL_CO_ are associated with poor survival rates and prognosis.^25^ Therefore, in this study, FVC, FEV1 and DL_CO_ were used for the respiratory function assessment.

#### Subtypes of ILD

Since ILD encompasses more than 200 parenchymal pulmonary disorders, we divided all the cases into subtypes, to facilitate the analysis. According to the classifications of Cottin *et al*,^26^ the subtypes contain idiopathic interstitial pneumonias, autoimmune ILDs, hypersensitivity pneumonitis, sarcoidosis and other ILDs. Beause IPF is the most widely studied and the most common type of ILD, we classified it as a dependent category to make the comparisons more detailed. Therefore, we included six subtypes in total.

### Data collection

We collected patients’ demographic data using a self-designed questionnaire, which was administered after 6MWT. The outcomes of the pulmonary function test and the NYHA functional classification were obtained from the patients’ medical records. The Borg scale was employed before and after walking. All the 6MWT-related outcomes were automatically collected using physiological parameters transmission management software during the 6-min module (Shenzhen zhongruiqi Electronic Technology Co.). Since the patients at the centre completed all the assessments and tests within 3 days of their admission, the outcomes of pulmonary function test, NYHA functional classification and 6MWT were obtained within the next 3 days.

### Analysis

We used the statistical package for the social sciences (SPSS) software V.21.0 (IBM Corporation) for data analysis. We used descriptive statistics to summarise the participants’ demographics, 6MWT outcomes, Borg grades, NYHA functional classification and pulmonary function indexes. Specifically, we described continuous variables as mean and SD, and categorical variables as frequency. After performing a check for normality, we used the paired t-test to assess the differences in SpO_2_ between each end of the minute. Analysis of variance was used to assess the differences of 6MWT-related outcomes across the subtypes of ILD. We performed the Pearson correlation analysis to identify the correlations between the 6MWT outcomes and other measurements. Statistical significance was set at p<0.05.

### Patient and public involvement

No patient involved.

## Results

### Demographics and characteristics of patients

We included 954 patients with ILD from July 2019 to August 2020. The average age of participants was 55.40 (SD=12.35) years (range 14–83 years). The sample included 510 (53.50%) men (table 1).

**Table 1:** The demographic and characteristic information of patients with interstitial lung disease (ILD) (n=954)

Variables	Categories	N (%)/Mean (SD)
Height (cm)	–	161.08 (8.00)
Weight (kg)	–	62.60 (10.60
BMI	–	24.07 (3.39)
Subclass of ILD	Autoimmune ILDs	277 (29.00）
	IIPs	195 (20.40）
	IPF	171 (17.90)
	Sarcoidosis	50 (5.20）
	Hypersensitivity pneumonitis	177 (18.60）
	Others ILDs	37 (3.90）
	Missing data	47 (4.90）

BMI, body mass index; IIPs, Idiopathic interstitial pneumonias; IPF, idiopathic pulmonary fibrosis.

### Features of 6MWT outcomes among patients with ILD

**Figure 1:**
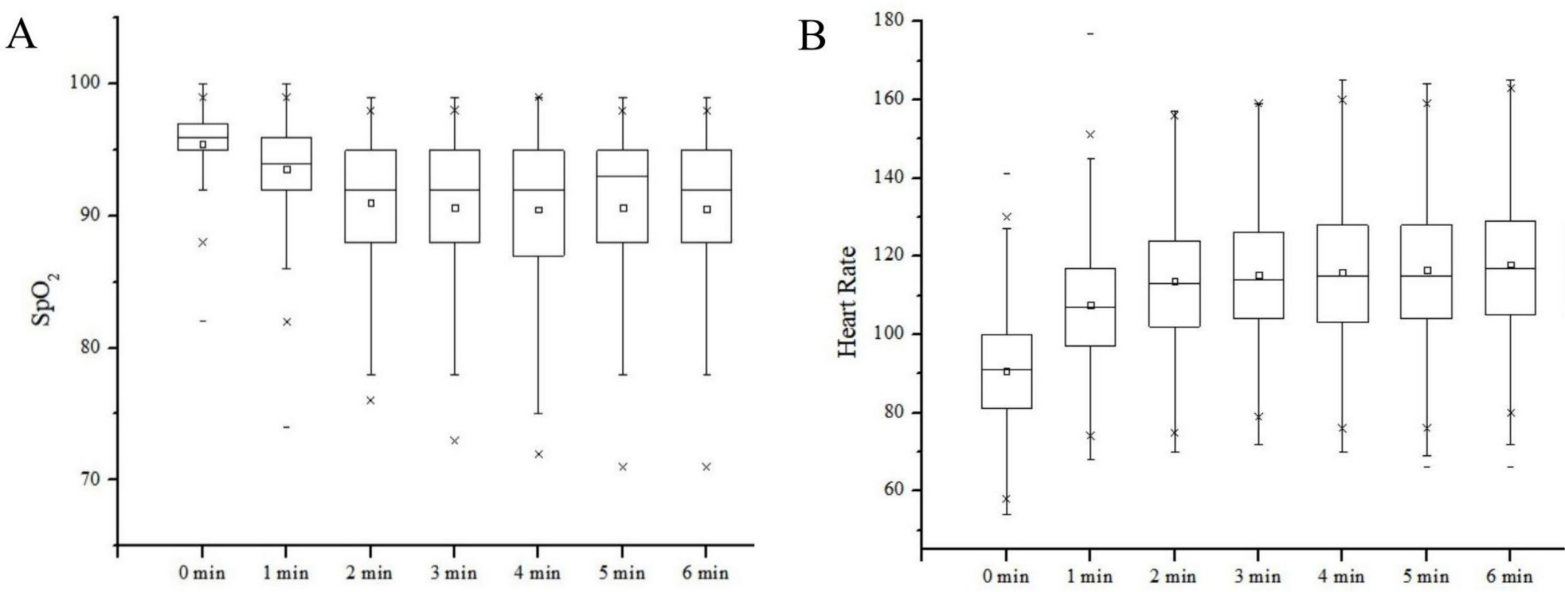
Description of SPO_2_ and heart rate (HR) in patients with interstitial lung disease during 6-min walk test.

**Figure 2:**
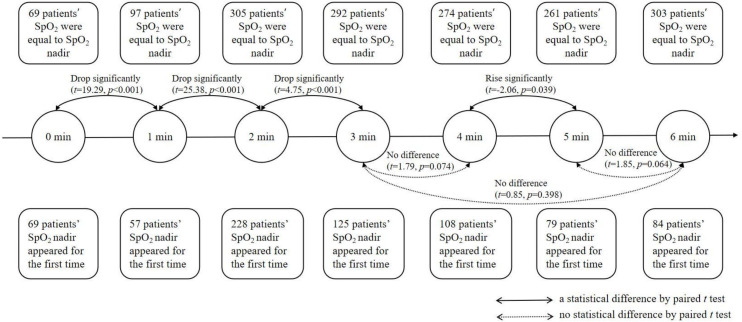
Statistical analysis chart of SPO_2_ in patients with interstitial lung disease within 6 min.

For 750 participants with valid data, the SpO_2_ nadir was higher than 80%, and for 524 patients (69.9%) the SpO_2_ nadir was higher than 88. Other details are shown in table 2. Figure 1 shows patients’ SpO_2_ and HR during 6MWT. SpO_2_ generally showed a downward trend, but increased slightly at the end of the fifth minute, whereas HR exhibited a sharp increase in the first 2 min and reached a peak before becoming steady. Paired t-test found three significant drops in the SpO_2_, which occurred at the first minute (t=19.29, p<0.001), the second minute (t=25.38, p<0.001) and the third minute (t=4.75, p<0.001). This was accompanied by a slightly significant rise at the fourth minute (t=−2.06, p=0.039). Figure 2 depicts the time point when SpO_2_ nadir appears at the first time and the occurrence of SpO_2_ nadir at each end of a minute over 6 min. The SpO_2_ nadir of 63.87% and 78.27% of the participants’ appeared at the end of the third and the fourth minute, respectively.

**Table 2:** The features of 6-min walk test (6MWT) outcomes among patients with interstitial lung disease

Items	N	Minimum	Maximum	Mean (SD)
Systolic blood pressure before 6MWT (mm Hg)	950	84	188	124.16 (17.26)
Systolic blood pressure after 6MWT (mm Hg)	734	88	242	138.46 (23.19)
Diastolic blood pressure before 6MWT (mm Hg)	950	50	128	77.64 (11.58)
Diastolic blood pressure after 6MWT (mm Hg)	734	49	167	82.45 (13.44)
Mean arterial pressure before 6MWT (mm Hg)	950	62.67	148.00	93.15 (12.16)
Mean arterial pressure before 6MWT (mm Hg)	734	66.67	182.00	101.12 (14.89)
Heart rate at 0 min (times/min)	750	50	141	90.97 (14.48)
Heart rate at 1 min (times/min)	749	65	177	107.69 (15.25)
Heart rate at 2 min (times/min)	749	70	192	113.66 (16.33)
Heart rate at 3 min (times/min)	749	68	199	115.43 (16.88)
Heart rate at 4 min (times/min)	749	70	201	116.08 (17.65)
Heart rate at 5 min (times/min)	749	66	195	116.49 (17.77)
Heart rate at 6 min (times/min)	749	66	193	117.97 (18.00)
Heart rate zenith (times/min)	750	70	201	121.38 (19.18)
Heart rate change (times/min)	749	−102.00	29.00	−27.07 (14.45)
SpO_2_ at 0 min (%)	750	82	100	95.49 (2.23)
SpO_2_ at 1 min (%)	748	74	100	93.61 (3.46)
SpO_2_ at 2 min (%)	749	65	99	91.03 (4.99)
SpO_2_ at 3 min (%)	749	65	99	90.62 (5.82)
SpO_2_ at 4 min (%)	749	58	99	90.49 (6.13)
SpO_2_ at 5 min (%)	749	61	99	90.65 (6.23)
SpO_2_ at 6 min (%)	748	56	99	90.54 (6.43)
SpO_2_ nadir (%)	750	56.00	99.00	89.09 (6.44)
SpO_2_ change (%)	748	−10.00	33.00	4.96 (5.57)
Distance (m)	953	53	999	457.28 (98.40)
Distance/ predicted distance (m)	953	12.49	175.1	84.74 (18.54)

Heart rate change and SpO_2_ change were the values at the beginning minus the values at the end of 6 min respectively.

SpO_2_, peripheral capillary oxygen saturation.

### Difference in 6MWT outcomes among subgroup of patients with ILD

Significant differences between the subtypes of ILD were found for 6MWD (*F*=2.44, p=0.033), SpO_2_ change (*F*=2.58, p=0.025), HR at 0 min (*F*=2.87, p=0.014), HR at end of 6 min (*F*=2.58, p=0.025) and HR zenith (*F*=2.64, p=0.022) (table 3).

**Table 3:** Difference between subgroups of interstitial lung disease (ILD) in 6-min walk test (6MWT) outcomes

Measures	Categories	N	Mean(SD)	*F*	P value
Borg score before 6MWT	Autoimmune ILDs	267	0.25 (0.54)	1.59	0.16
	IIPs	192	0.18 (0.50)		
	IPF	167	0.28 (0.51)		
	Sarcoidosis	49	0.18 (0.39)		
	Others ILDs	37	0.35 (0.63)		
	Hypersensitivity pneumonitis	174	0.31 (0.59)		
Borg score after 6MWT	Autoimmune ILDs	267	1.25 (1.15)	0.97	0.433
	IIPs	192	1.32 (1.21)		
	IPF	167	1.37 (1.23)		
	Sarcoidosis	49	1.04 (0.98)		
	Others ILDs	36	1.14 (1.17)		
	Hypersensitivity pneumonitis	174	1.38 (1.15)		
6MWD (m)	Autoimmune ILDs	277	452.69 (96.25)	2.44	0.033
	IIPs	195	466.07 (96.86)		
	IPF	171	440.70 (87.85)		
	Sarcoidosis	50	479.36 (91.25)		
	Others ILDs	37	482.30 (116.70)		
	Hypersensitivity pneumonitis	177	453.48 (107.31)		
6MWD/predicted 6MWD	Autoimmune ILDs	277	0.84 (0.18)	0.84	0.521
	IIPs	195	0.85 (0.19)		
	IPF	171	0.87 (0.18)		
	Sarcoidosis	50	0.87 (0.17)		
	Others ILDs	37	0.84 (0.23)		
	Hypersensitivity pneumonitis	177	0.84 (0.19)		
SpO2 at the 0 min (%)	Autoimmune ILDs	219	95.75 (1.98)	2.14	0.059
	IIPs	143	95.15 (2.53)		
	IPF	143	95.14 (2.33)		
	Sarcoidosis	45	95.40 (1.76)		
	Others ILDs	27	95.37 (3.12)		
	Hypersensitivity pneumonitis	135	95.70 (2.21)		
SpO_2_ at the end of 6 min (%)	Autoimmune ILDs	218	90.78 (6.07)	2.14	0.059
	IIPs	143	91.07 (5.74)		
	IPF	143	88.91 (6.49)		
	Sarcoidosis	45	90.89 (6.41)		
	Others ILDs	27	91.07 (5.86)		
	Hypersensitivity pneumonitis	134	90.07 (7.85)		
SpO2 nadir (%)	Autoimmune ILDs	219	89.20 (6.01)	1.421	0.215
	IIPs	143	89.60 (5.52)		
	IPF	143	87.86 (6.56)		
	Sarcoidosis	45	88.89 (6.47)		
	Others ILDs	27	90.15 (5.82)		
	Hypersensitivity pneumonitis	135	88.59 (8.08)		
SpO_2_ change (%)	Autoimmune ILDs	218	4.98 (5.52)	2.58	0.025
	IIPs	143	4.08 (5.04)		
	IPF	143	6.23 (5.05)		
	Sarcoidosis	45	4.51 (5.88)		
	Others ILDs	27	4.30 (3.61)		
	Hypersensitivity pneumonitis	134	5.63 (6.93)		
HR at the 0 min (times/min)	Autoimmune ILDs	219	92.21 (15.09)	2.87	0.014
	IIPs	143	91.12 (12.75)		
	IPF	143	87.15 (13.80)		
	Sarcoidosis	45	88.44 (14.46)		
	Others ILDs	27	91.81 (13.76)		
	Hypersensitivity pneumonitis	135	92.40 (15.61)		
HR at the end of 6 min (times/min)	Autoimmune ILDs	219	119.45 (17.65)	2.58	0.025
	IIPs	143	118.76 (18.63)		
	IPF	143	113.05 (15.41)		
	Sarcoidosis	45	118.33 (20.99)		
	Others ILDs	27	116.30 (16.22)		
	Hypersensitivity pneumonitis	135	118.57 (18.77)		
HR zenith (times/min)	Autoimmune ILDs	219	122.29 (18.61)	2.64	0.022
	IIPs	143	121.86 (19.58)		
	IPF	143	115.90 (15.79)		
	Sarcoidosis	45	124.09 (23.88)		
	Others ILDs	27	122.22 (19.73)		
	Hypersensitivity pneumonitis	135	122.07 (20.53)		
HR change (times/min)	Autoimmune ILDs	219	−27.23 (14.95)	0.8	0.547
	IIPs	143	−27.64 (15.23)		
	IPF	143	−25.90 (12.03)		
	Sarcoidosis	45	−29.89 (14.32)		
	Others ILDs	27	−24.48 (12.92)		
	Hypersensitivity pneumonitis	134	−26.54 (14.52)		

HR, heart rate; IIPs, Idiopathic interstitial pneumonias; IPF, idiopathic pulmonary fibrosis; 6MWD, 6-min walk distance; SpO_2:_ peripheral capillary oxygen saturation.

### Correlation between the outcomes of 6MWT and cardiopulmonary function

SpO_2_ was generally positively correlated to cardiopulmonary function, whereas the HR and Borg scale were negatively correlated. Specifically, the NYHA grade strongly correlated with 6MWD (*r=*0.82, p*<*0.01). The 6MWD/predicted 6MWD had the highest correlation coefficient with FVC (*r=*0.30, p*<*0.01) and FEV_1_ (*r=*0.30, p*<*0.01). SpO_2_ at the end of 3 min had the strongest correlation to DL_CO_ (*r=*0.41, p*<*0.01) (table 4).

**Table 4:** Correlations between 6-min walk test (6MWT) outcomes and cardiopulmonary function

Outcomes of 6MWT		NYHA	MAP before 6MWT	MAP after 6MWT	MAP change	FVC	FEV_1_	DL_CO_
6MWD (m)	*r*	0.82**	0.07*	0.09*	0.08*	0.24**	0.17**	0.26**
	n	751	949	733	733	846	846	806
6MWD/predicted 6MWD	*r*	0.64**	0.09**	0.14**	0.09*	0.30**	0.30**	0.28**
	n	751	949	733	733	846	846	806
SpO_2_ at 0 min (%)	*r*	0.29**	0.03	0.01	−0.02	0.18**	0.17**	0.26**
	n	750	746	732	732	644	644	604
SpO_2_ at end of 1 min (%)	*r*	0.27**	0.02	0.01	0.01	0.24**	0.21**	0.31**
	n	748	744	730	730	642	642	602
SpO_2_ at end of 2 min (%)	*r*	0.28**	0.05	0.03	−0.01	0.27**	.23**	.37**
	n	749	745	731	731	643	643	603
SpO_2_ at end of 3 min (%)	*r*	0.29**	0.047	0.034	0.00	0.29**	0.24**	0.41**
	n	749	745	731	731	643	643	603
SpO_2_ at end of 4 min (%)	*r*	0.27**	0.03	0.03	0.01	0.27**	0.23**	0.40**
	n	749	745	731	731	643	643	603
SpO_2_ at end of 5 min (%)	*r*	0.27**	0.03	0.03	0.01	0.27**	0.23**	0.39**
	n	749	745	731	731	643	643	603
SpO_2_ at end of 6 min (%)	*r*	0.26**	0.03	0.02	0.01	0.27**	0.22**	0.37**
	n	748	744	730	730	642	642	602
SpO_2_ nadir (%)	*r*	0.27**	0.05	0.04	0.01	0.28**	0.24**	0.38**
	n	750	746	732	732	644	644	604
SpO_2_ change (%)	*r*	−0.18**	−0.02	−0.02	−0.02	−0.24**	−0.19**	−0.32**
	n	748	744	730	730	642	642	602
Borg scale at 0 min	*r*	−0.23**	−0.02	0.02	0.05	−0.12**	−0.06	−0.13**
	n	727	926	714	714	829	829	791
Borg scale at the end	*r*	−0.25**	−0.05	0.00	0.07	−0.15**	−0.11**	−0.19**
	n	726	925	713	713	828	828	790
HR at 0 min (times/min)	*r*	−0.02	0.05	0.03	−0.01	−0.18**	−0.21**	−0.10*
	n	750	746	732	732	644	644	604
HR at end of 1 min (times/min)	*r*	0.16**	0.09**	0.106**	0.04	−0.08	−0.12**	0.00
	n	749	745	731	731	643	643	603
HR at end of 2 min (times/min)	*r*	0.18**	0.09*	0.13*	0.07	−0.08*	−0.11**	−0.02
	n	749	745	731	731	643	643	603
HR at end of 3 min (times/min)	*r*	0.18**	0.12**	0.16**	0.09*	−0.08	−0.11**	−0.01
	n	749	745	731	731	643	643	603
HR at end of 4 min (times/min)	*r*	0.19**	0.10**	0.14**	0.08*	−0.06	−0.11**	−0.03
	n	749	745	731	731	643	643	603
HR at end of 5 min (times/min)	*r*	0.22**	0.11**	0.16**	0.10**	−0.05	−0.09*	−0.02
	n	749	745	731	731	643	643	603
HR at end of 6 min (times/min)	*r*	0.24**	0.10**	0.16**	0.11**	−0.04	−0.09*	−0.02
	n	749	745	731	731	643	643	603
HR zenith (times/min)	*r*	0.19**	0.10**	0.12**	0.06	−0.03	−0.09*	0.01
	n	750	746	732	732	644	644	604
HR change (times/min)	*r*	−0.32**	−0.08*	−0.17*	−0.15**	−0.14**	−0.11**	−0.09*
	n	749	745	731	731	643	643	603

**P*<*0.01, *p<0.05.

DLCO, diffusing capacity of the lungs for carbon monoxide; FEV_1_, forced expiratory volume in the first second; FVC, forced vital capacity; HR, heart rate; MAP, mean arterial pressure; 6MWD, 6-min walk distance; NYHA, New York Heart Association; SpO_2_, peripheral capillary oxygen saturation.

## Discussion

This study described identified the correlation between 6MWT outcomes and cardiopulmonary function and compared the difference between subtypes of ILD on 6MWT outcomes. We found that the HR and SpO_2_ did not increase or decrease uniformly during walking. For approximately 10% of the patients, the SpO_2_ nadir was lower than 80%, but they completed the test. Besides, SpO_2_ nadir appeared at the end of the fourth minute for approximately 80% of patients. Therefore, 6MWD and SpO_2_ had the strongest correlation with heart function and lung function of ILD, respectively. Moreover, group comparisons revealed that the 6MWD and SpO_2_ change were more distinguishing for the subgroups of ILD.

Compared with previous studies, the average 6MWD in our study was 457.28 m, which was moderate.^27 28^ HR increased continuously and SpO_2_ decreased, with a slight rise in the fifth minute. However, the results of a previous study showed a slight increase in the fourth minute and a sharp drop in SpO_2_.^29^ Since our study had a bigger sample size, the average 6MWD and tendency of SpO_2_ were more representative. According to the standard, 6MWT should be terminated when SpO_2_ falls below 80%.^30^ When SpO_2_ was less than 88%, it was considered as a significant desaturation, and patients were recommended to take an oxygen supplement.^31–33^ Without oxygen supplements in our study, SpO_2_ nadir were lower than 88% and 80% for 30.1% and 9.3% of the patients, respectively. They all completed 6MWT without any chest pain, leg cramps, unsteady gait, diaphoresis or a pale/ashen appearance, experiencing breathlessness, or reporting being too tired to continue. Our findings indicated that it is unwarranted to stop 6MWT when patients with ILD only experience desaturation without other indications of termination, which corroborate the findings of Afzal *et al*.^34^ The SpO_2_ nadir is an essential outcome of 6MWT, and our research revealed that for 63.87% and 78.27% of the participants, SpO_2_ nadir appeared at the end of the third and fourth minute respectively. Oxygen titration is generally performed with 6MWT to determine the oxygen flow that prevents oxygen saturation from falling below 88%, measured using pulse oximetry (SpO_2_). According to Giovacchini *et al*,^35^ after a certain dose of oxygen is administered, the patients’ SpO_2_ should exceed 88% and be stable for 3 min. In our study, we found that the SpO_2_ nadir for approximately 80% of the patients had appeared at the end of the fourth minute; hence, we strongly recommend that oxygen titration should be for 4 min.

Garin *et al* did not find significant differences between IPF and systemic sclerosis-associated ILD on 6MWD and dyspnoea,^36^ while Someya and Mugii found that patients with IPF had lower SpO_2_ and higher Borg score than patients with dermatomyositis.^37^ We observed no significant differences between the subtypes for Borg score and SpO_2_ after walking. Since previous studies merely compared two different subgroups of ILD, our results were more comprehensive and reliable. Contrary to dyspnoea and SpO_2_ after walking, we found significant differences between groups on 6MWD and SpO_2_ change. Therefore, 6MWD and SpO_2_ change was the more distinguishing outcomes for subtypes of ILD. Although the HR at the 0 min, end of 6 min and HR zenith showed significant differences between the subtypes, this finding was unclear because baseline HR showed differences before walking. Therefore, future studies in another population or multicentre may reinforce the findings.

Similar to previous studies, 6MWD and SpO_2_ positively correlated with cardiopulmonary function outcomes such as NYHA, FVC, FEV_1_ and DL_CO_,^5 8–10^ while Borg score was negatively correlated.^30^ Compared with 6MWD and SpO_2_, the patients’ HR had a weaker positive correlation to cardiopulmonary function outcomes. Hence, SpO_2_ and distance were more valuable than HR in predicting the patients’ cardiopulmonary function and degree of dyspnoea. In a study, 6MWD was more correlated to DL_CO_ than SpO_2_ 27; however, our result was to the contrary—lower FVC and DL_CO_ were associated with poor prognosis and high mortality.^16 38–40^ Nevertheless, the DL_CO_ level was more valuable than FVC, as it captured the combined impact on the pulmonary reserve of IPF, emphysema and pulmonary hypertension.^41^ Since SpO_2_ had the strongest correlation with DL_CO_, we recommend clinical practitioners to monitor the SpO_2_ of patients with ILD. Besides, the most valuable SpO_2_ time point remains unclear. A previous study revealed that SpO_2_ nadir and SpO_2_ change had the same degree of correlation with DL_CO_,^42^ and another study also highlighted the critical predictive value of the SpO_2_ nadir.^43^In contrast, our findings illustrated that SpO_2_ at the end of the third minute was more predictive than the SpO_2_ nadir and SpO_2_ change in DL_CO_. Although 6MWD/predicted 6MWD had a higher correlation to FEV_1_ and FVC than SpO_2_ at the end of the third minute, 6MWD was more susceptible to factors such as age, sex, shorter corridor and inappropriate walking shoes.^44–46^ Furthermore, DL_CO_ was considered more critical than FEV_1_ and FVC for ILD. Hence, the third-minute SpO_2_ can be an alternative to predict lung function in patients with ILD.

### Limitations and future research directions

This study had several limitations. First, we did not conduct the second 6MWT for patients, and so the measured distance might not be the longest potential distance. Second, 25% of the values regarding some non-critical variables were missing, which might introduce selection bias and affect the validity and representativeness of the results. Replication in another population or a multicentric study could reinforce the findings. Third, lack of follow-up on patients’ prognosis and mortality hindered the prediction of 6MWT outcomes on the long-term clinical outcomes. Future research is required to explore the association between the outcomes of 6MWT and long-term prognosis.

## Conclusions

Despite the above limitations, this study showed that increased HR and decreased SpO_2_ during the 6MWT do not change uniformly. Approximately 10% of the patients, whose SpO_2_ was less than 80%, completed 6MWT without any discomfort indicated. Hence, it is unwarranted to halt 6MWT when patients with ILD experience only desaturation, without other indications of termination. SpO_2_ nadir appeared at the end of the fourth minute for approximately 80% of the patients, which provides an important evidence regarding oxygen titration, that is, it is better to maintain SpO_2_ above 88% for 4 min. Besides, the third-minute SpO_2_ can be an alternative to predict patients’ lung function. Conclusively, 6MWD and SpO_2_ change showed significant differences between the subtypes of ILD, which indicated that they were more distinguishing for the subtypes of ILD.

## Supplementary Material

Reviewer comments

Author's manuscript

## Data Availability

Data are available upon reasonable request. Data were available upon reasonable request from Xiaobing Wu (wuxiaobing_gz@163.com)
