# Supplementary material for: Features and predictive value of 6-min walk test outcomes in interstitial lung disease: an observation study using wearable monitors
Source: BMJ Open. 2022 Jun 15;12(6):e055077. doi: 10.1136/bmjopen-2021-055077 (PMC9204441; doi:10.1136/bmjopen-2021-055077)
Supplement: Author's manuscript [file bmjopen-2021-055077.draft_revisions.pdf]

BMJ Open

BMJ Open is committed to open peer review. As part of this commitment we make the peer review history of every article we publish publicly available.

When an article is published we post the peer reviewers' comments and the authors' responses online. We also post the versions of the paper that were used during peer review. These are the versions that the peer review comments apply to.

The versions of the paper that follow are the versions that were submitted during the peer review process. They are not the versions of record or the final published versions. They should not be cited or distributed as the published version of this manuscript.

BMJ Open is an open access journal and the full, final, typeset and author-corrected version of record of the manuscript is available on our site with no access controls, subscription charges or pay-per-view fees (http://bmjopen.bmj.com).

If you have any questions on BMJ Open's open peer review process please email info.bmjopen@bmj.com

Detailed Features and Predictive Value of 6MWT Outcomes in Interstitial Lung Disease: An Observation Study by Wearable Monitor

Journal:	*BMJ Open*
Manuscript ID	bmjopen-2021-055077
Article Type:	Original research
Date Submitted by the Author:	03-Jul-2021

Complete List of Authors:	LI, Jiaying; The University of Hong Kong, School of Nursing<br>LI, Xiaoyan; First Affiliated Hospital of Guangzhou Medical College, Guangzhou Institute of Respiratory Health<br>DENG, Miaozhen; First Affiliated Hospital of Guangzhou Medical College, Guangzhou Institute of Respiratory Health<br>LIANG, Xinyin; First Affiliated Hospital of Guangzhou Medical College, Guangzhou Institute of Respiratory health<br>WEI, Huiqun; First Affiliated Hospital of Guangzhou Medical College, Guangzhou Institute of Respiratory Health<br>Wu, Xiaobing; First Affiliated Hospital of Guangzhou Medical College, Department of Internal Medicine

Keywords:	Interstitial lung disease < THORACIC MEDICINE, Rehabilitation medicine < INTERNAL MEDICINE, RESPIRATORY MEDICINE (see Thoracic Medicine)

I, the Submitting Author has the right to grant and does grant on behalf of all authors of the Work (as defined in the below author licence), an exclusive licence and/or a non-exclusive licence for contributions from authors who are: i) UK Crown employees; ii) where BMJ has agreed a CC-BY licence shall apply, and/or iii) in accordance with the terms applicable for US Federal Government officers or employees acting as part of their official duties; on a worldwide, perpetual, irrevocable, royalty-free basis to BMJ Publishing Group Ltd ("BMJ") its licensees and where the relevant Journal is co-owned by BMJ to the co-owners of the Journal, to publish the Work in this journal and any other BMJ products and to exploit all rights, as set out in our licence.

The Submitting Author accepts and understands that any supply made under these terms is made by BMJ to the Submitting Author unless you are acting as an employee on behalf of your employer or a postgraduate student of an affiliated institution which is paying any applicable article publishing charge ("APC") for Open Access articles. Where the Submitting Author wishes to make the Work available on an Open Access basis (and intends to pay the relevant APC), the terms of reuse of such Open Access shall be governed by a Creative Commons licence – details of these licences and which Creative Commons licence will apply to this Work are set out in our licence referred to above.

Other than as permitted in any relevant BMJ Author's Self Archiving Policies, I confirm this Work has not been accepted for publication elsewhere, is not being considered for publication elsewhere and does not duplicate material already published. I confirm all authors consent to publication of this Work and authorise the granting of this licence.

For peer review only

Title Page

Author and Co-author's name

Jiaying LI[1], Xiaoyan LI[2], Miaozhen DENG[2], Xinyin LIANG[2], Huiqun WEI[2], Xiaobing WU[2]

Information of corresponding author

Xiaobing WU, First Affiliated Hospital of Guangzhou Medical College. No.151 Yanjiang West Road, Yuexiu District, Guangzhou, Guangdong, China, wuxiaobing_gz@163.com, +86 13711663164

Information of co-authors

Jiaying LI, The University of Hong Kong, School of Nursing, Hong Kong, China

Xiaoyan LI, First Affiliated Hospital of Guangzhou Medical College, Guangzhou, China

Miaozhen DENG: First Affiliated Hospital of Guangzhou Medical College, Guangzhou, China

Xinyin LIANG: First Affiliated Hospital of Guangzhou Medical College, Guangzhou, China

Huiqun WEI: First Affiliated Hospital of Guangzhou Medical College, Guangzhou, China

Keywords: 6MWT, interstitial lung disease, predictive value, cardiopulmonary function

Word count: 3078

For peer review only

Detailed Features and Predictive Value of 6MWT Outcomes in Interstitial Lung Disease: An Observation Study by Wearable Monitor

Abstract Objectives This study aims to continuously and comprehensively obtain and describe the features of 6MWT outcomes in ILD patients, to identify the best predictive indicator for cardiopulmonary function, and to compare the difference of 6MWT among various sub-types of ILD.

Methods The outcomes of 6MWT were accurately collected by wearable monitors. NYHA classification and pulmonary function tests were utilized to evaluate cardiopulmonary function.

Results Heart rate present a continuous upward trend, while SpO_2 showed an overall downward with a slight increase at the fifth minute. There is no difference between the third and sixth minute in SpO_2 (t=0.85, p=0.398). 680 patients' (90.7%) SpO_2 nadir were higher than 80 completed the 6MWT without any discomfort, 63.87% and 78.27% participants' SpO_2 nadir appeared at the end of the third and fourth minute respectively. No significant difference was found in all outcomes of 6MWT among the sub-group of ILDs (p>0.05). SpO_2 at the end of 3 min had the strongest correlation to patients' FVC (r=0.29, p<0.01), FEV_1 (r=0.24, p<0.01) and DL_{CO} (r=0.41, p<0.01)

Conclusion Most patients' SpO_2 nadir appears at the end of the fourth minute, which provided evidence for oxygen titration that it's better to maintain SpO_2 above 88% for four minutes. Besides, the third-minute SpO_2 is most valuable and is recommended as a clinical anchor in a pulmonary rehabilitation program. No difference in sub-type ILDs also helps clinical practitioners a better interpretation of 6MWT outcomes.

Keywords: 6MWT, interstitial lung disease, predictive value, cardiopulmonary function

Strengths of this study

- This is the first study with large sample focus on 6MWT outcomes in ILD.
- This is the first study compare the 6MWT outcomes between various type of ILD.
- This study is by far the most detailed description of the results of 6MWT outcomes in ILD.

Limitations of this study

- Lacking of follow-up study limits the predictive value of 6MWT outcomes to current cardiopulmonary function

Background

Interstitial lung disease (ILD) is a group of more than 200 kinds of failure diseases characterized by pulmonary inflammation with or without fibrosis.[1,2] Patients diagnosed with ILD mostly have

For peer review only

dyspnea, decreased exercise tolerance.[3] The six-minute walk test (6MWT) is wildly used for assessing patients' performance ability in different cardiopulmonary-related diseases, which gives essential practiced outcomes that are cannot be obtained in standardized pulmonary function testing.[4]

By far, limited studies have focused on the outcomes of 6MWT in ILD and mainly focus on idiopathic pulmonary fibrosis (IPF). The research to date has tended to center on the six-minute walk distance (6MWD) and percutaneous oxygen saturation (SpO_2). Specifically, 6MWD and desaturation have been proved to have an association with mortality in patients with IPF,[5,6] and 6MWD was also an independent factor of IPF patients' physical activity.[7] In addition to mortality and physical activity, 6MWD has a positive association with objective lung function index, including Forced vital capacity (FVC) % predicted, Diffusing capacity of the lungs for carbon monoxide (DLco) % predicted,[5,8,9] Forced Expiratory Volume in the first second (FEV_{1})),[10] and health-related quality of life (HRQL),[8,9] while it was confirmed to be a negative indicator for dyspnea.[8,9, 11,12] Furthermore, the occurrence of desaturation when SpO_2 is less than 88% and changes in SpO_2 during the test have been identified as an indicator of patients' mortality with IPF.[13,14]

As we all know, the 6MWT contains multiple outcomes that are not limited to 6MWD and SpO_2. It comprises walk distance, heart rate, blood pressure, blood oxygen saturation, and dyspnea according to the Borg scale.[15] Although many outcomes have been proved to have predictive value, which indicator at which point of time is the most valuable predictor for patients' cardiopulmonary function remains unclear. What's more, in addition to IPF, whether the results of patients with other types of ILD are consistent has not been confirmed. Since several studies highlighted the importance to find out the most prognostic value outcome of 6MWT[6,16] and measurements of SpO_2 throughout the 6MWT has been recommended by the 2014 ERS/ATS technical standard,[17] the wearable monitor is a feasible approach to collect multiple data simultaneously and make a better comparison between each 6MWT outcomes on their predictive value.

Hence, to provide a better interpretation of 6MWT outcomes for clinical medical staff, and to help those that unable to continuously monitor the indicators of 6MWT to easily obtain the most valuable data, this study aims to (1) describe the detailed features of 6MWT outcomes comprehensively and continuously by the wearable monitor with a large sample size in ILD patients that not limited to IPF patients, including heart rate (HR) and SpO_2 at the beginning, end, and end of each minute, blood pressure at the beginning and end, and distance. (2) explore the correlation

between the outcomes of 6MWT and patients' cardiopulmonary function. (3) find out whether there are differences in the 6MWT outcomes among different sub-classification of ILD patients.

Methods

Design

An observation study by using a wearable monitor was conducted in this study.

Patients

Inclusion criteria for participants: (1) diagnosed with interstitial lung disease; (2) Patients' condition was feasible to conduct 6MWT; (3)Patients were informed and voluntarily participated in the study. Exclusion criteria: (1) patients who were unwilling to participate were excluded; (2) Patients' condition was unsuitable for 6MWT. The unsuitable condition consists of the following. Patients who had serious limitations to walk, such as joint restrictions or other critical diseases that make 6MWT not feasible. Besides, patients who have myocardial infarction in five days, unstable angina, syncope, symptomatic arrhythmia, or severe aortic stenosis, decompensated heart failure among other unstable medical issues were unsuitable for 6MWT.[17]

Instruments

Demographics information questionnaire

Demographics information related to age, height, weight, BMI, and gender were collected by a self-design questionnaire.

Borg scale

The Borg Rating of Perceived Exertion (RPE) scale was developed by Borg,[18] which was wildly used for measuring patients' effort and exertion, breathlessness, and fatigue during physical work. According to the standards,[19] a higher score indicated a severer level of exertion.

NYHA functional class

The New York Heart Association (NYHA) Classification was created and revised as a critical criterion for a comprehensive cardiac diagnosis.[20] in this study, the classification of patients' NYHA is according to patient symptoms which are arranging from no symptoms with ordinary physical activity (Class I) to symptoms at rest and increased discomfort with any physical activity (Class IV).[21]

Outcomes of 6MWT

According to Enright's recommendations,[22] the primary measurement in our study is 6MWD,

For peer review only

and the secondary measures consist of fatigue and dyspnea which is measured by a modified Borg. A portable oximeter was utilized for monitoring patients' arterial oxygen saturation. In addition to this, we also recorded the heart rate at each end of the minute, and blood pressure before and after the test.

Pulmonary function index

Restricted ventilation dysfunction and diffusion dysfunction are the main symptoms of interstitial lung disease. The FEV_1/FVC increase was consistent with typically restricted ventilation dysfunction. Since previous studies have proven that reduction in Forced Vital Capacity (FVC) and Diffusion Capacity for Carbon Monoxide of the Lung (DL_{CO}) are associated with poor survival rates and prognosis.[23] Therefore, FVC, forced expiratory volume in one second (FEV_1) and DL_{CO} is planned to record in this study for a representative for patients' pulmonary function index.

Subclass of ILD

Since ILD encompasses more than 200 parenchymal pulmonary disorders, all the cases we concluded in this study were classified according to Cottin *et al.*,[24] which contains IIPs, Autoimmune ILDs, Hypersensitivity pneumonitis, Sarcoidosis, and other ILDs.

Data collection

All the data in this study were automatically collected by physiological parameters transmission management software in 6 minutes module (Shenzhen zhongruiqi Electronic Technology Co., Ltd) from July 2019 to August 2020. Each patient was given 6MWT once only. The 6MWT was conducted without oxygen therapy support.

Analysis

SPSS 21.0 (IBM Corporation, Armonk, New York, USA) was utilized for data analysis. For the description of participants' demographic profiles, 6MWT outcomes, Borg grades, NYHA functional class, and pulmonary related function. All the variables were reported by descriptive statistics, and mean (M) and standard deviations (SD) were utilized for continuous variables. Normality checks were performed for all variables, and normality transformation was carried out when needed. The differences of SpO_2 between each end of the minute were compared by Paired Sample T-test. To identify the difference between each 6MWT outcome and sub-classification diseases, t-test or ANOVA analyses were conducted. Pearson correlation analysis was used to analyze the correlation between the 6MWT outcomes and other measurements.

Patient and Public Involvement

For peer review only

No patient involved.

Results

3.1 Demographic and characteristic profiles of participants

954 patients with ILD were concluded from 2019 to 2020 in this study. The age of respondents was between 14 to 83 with an average age of 55.40 (12.35). Other patients' demographic information was detailed in **Table 1**.

Table 1 the demographic and characteristic information of ILD patients (n=954)

Items	Category	*n(%)*	*Mean(SD)*
Age	-	-	55.40 (12.35)
Height (cm)	-	-	161.08 (8.00)
Weight (kg)	-	-	62.60 (10.60
Body mass index (BMI)	-	-	24.07 (3.39)
Gender	Male	510（53.50）	-
	Female	444（46.50）	-
Degree of dyspnea	0	739（77.50）	-
	1	149（15.60）	-
	2	39（4.10）	-
	3	2（0.20）	-
	Missing data	25 (2.6%)	-
NYHA functional class	1	38（4.00）	-
	2	93（9.70）	-
	3	190（19.90）	-
	4	430（45.10）	-
	Missing data	203 (21.3)	-
Subclass of ILD	Autoimmune ILDs	277（29.00）	-
	IIPs	366（38.40）	-
	Sarcoidosis	50（5.20）	-

For peer review only

Hypersensitivity pneumonitis	177（18.60）	-
Others ILDs	37（3.90）	
Missing data	47（4.90）	-

3.2 The features of 6MWT outcomes among ILD patients

In 750 valid data, 680 patients (90.7%) SpO_2 nadir was higher than 80, and 524 patients (69.9%) has a higher SpO_2 nadir than 88. Details are contained in **Table 2.** Furthermore, the tendency of patients' SpO_2 and HR during 6MWT is present in **Figure 1**. SpO_2 generally showed a downward trend, but slightly increased at the end of the fifth minute, whereas HR saw a sharp increase in the first two minutes and reached a peak before it remained steady. Specifically, features of SpO_2 by paired sample *t* test proved three significant drops of SpO_2 at fist minute(t=19.29, p<0.001), second minute (t=25.38, p<0.001), and third minute (t=4.75, p<0.001), but insignificant difference were founded between the third minute and the sixth minute(t=0.85, p=0.398), between the third and fourth minutes (t=1.79, p=0.074), and between fifth-minute and six-minute (t=1.85, p=0.064). what's more, a slight but significant increase took place at the end of the fourth minute (t=-2.06, p=0.039). In addition, **Figure 2** showed the distribute and the time when SpO_2 nadir appears in chronological order. 63.87% and 78.27%of participants' SpO_2 nadir appeared at the end of the third and the fourth minute respectively.

Table 2 the features of 6MWT outcomes among ILD patients

Items	n	Minimum	Maximum	Mean (SD)
Systolic blood pressure before 6MWT	950	84	188	124.16 (17.26)
Systolic blood pressure after 6MWT	936	50	242	125.08 (33.09)
Diastolic blood pressure before 6MWT	748	50	128	77.97 (11.96)
Diastolic blood pressure after 6MWT	734	49	167	82.45 (13.44)
Heart rate at beginning	750	50	141	90.97 (14.48)
Heart rate at 1 min	749	65	177	107.69 (15.25)
Heart rate at 2 min	749	70	192	113.66 (16.33)
Heart rate at 3 min	749	68	199	115.43 (16.88)
Heart rate at 4 min	749	70	201	116.08 (17.65)
Heart rate at 5 min	749	66	195	116.49 (17.77)

For peer review only

Heart rate at 6 min	749	66	193	117.97 (18.00)
Heart rate zenith	750	70	201	121.38(19.18)
Heart rate change	749	-102.00	29.00	-27.07(14.45)
Heart rate at end	952	42	193	118.19 (18.38)
SpO_2 at beginning	750	82	100	95.49 (2.23)
SpO_2 at 1 min	748	74	100	93.61 (3.46)
SpO_2 at 2 min	749	65	99	91.03 (4.99)
SpO_2 at 3 min	749	65	99	90.62 (5.82)
SpO_2 at 4 min	749	58	99	90.49 (6.13)
SpO_2 at 5 min	749	61	99	90.65 (6.23)
SpO_2 at 6 min	748	56	99	90.54 (6.43)
SpO_2 at end	952	56	99	90.42 (6.27)
SpO_2 nadir	750	56.00	99.00	89.09(6.44)
SpO_2 change	748	-10.00	33.00	4.96(5.57)
Distance	953	53	999	457.28 (98.40)
Distance/ predicted distance	953	12.49	175.1	84.74 (18.54)

Heart rate change and SpO_2 change were the value at the beginning minus the value at the end of six minutes respectively.

For peer review only

3.3 Difference in 6MWT outcomes among sub-group of ILD patients

As shown in **Table 3**, no significant difference was found in NYHA functional class, Borg scale at the beginning and end of the 6MWT, 6MWD, SpO_2 at the end of 6MWT, HR at the beginning and end of 6MWT among sub-group of ILDs ($p>0.05$).

Table 3. difference between sub-group of ILD diseases in 6MWT outcomes

**Measures**	**Categories**	**n**	**Mean(SD)**	χ^2	***p***
Borg scale at the beginning of the 6MWT	Autoimmune ILDs	267	0.25 (0.54)	1.22	0.302
	IIPs	359	0.23 (0.51)		
	Sarcoidosis	49	0.18 (0.39)		
	Others ILDs	37	0.35 (0.63)		
	Hypersensitivity pneumonitis	174	0.31 (0.59)		

Borg scale at the end of the 6MWT	Autoimmune ILDs	267	1.25 (1.15)	1.19	0.314
	IIPs	359	1.34 (1.22)		
	Sarcoidosis	49	1.04 (0.98)		
	Others ILDs	36	1.14 (1.17)		
	Hypersensitivity pneumonitis	174	1.38 (1.15)		
6MWD	Autoimmune ILDs	277	452.69 (96.25)	1.51	0.196
	IIPs	366	454.22 (93.50)		
	Sarcoidosis	50	479.36 (91.25)		
	Others ILDs	37	482.30 (116.70)		
	Hypersensitivity pneumonitis	177	453.48 (107.31)		
SpO_2 at the beginning of 6MWT	Autoimmune ILDs	277	95.78 (2.00)	3.68	0.006
	IIPs	366	95.11 (2.40)		
	Sarcoidosis	50	95.54 (1.75)		
	Others ILDs	37	95.38 (3.12)		
	Hypersensitivity pneumonitis	177	95.60 (2.27)		
SpO_2 at the end of 6MWT	Autoimmune ILDs	277	90.84 (5.75)	2.04	0.087
	IIPs	366	89.68 (6.20)		
	Sarcoidosis	50	91.34 (6.24)		
	Others ILDs	37	91.19 (5.68)		
	Hypersensitivity pneumonitis	176	89.94 (7.38)		
SpO_2 nadir	Autoimmune ILDs	219	89.20 (6.01)	0.49	0.745
	IIPs	286	88.73 (6.11)		
	Sarcoidosis	45	88.89 (6.47)		
	Others ILDs	27	90.15 (5.82)		
	Hypersensitivity pneumonitis	135	88.59 (8.08)		
SpO_2 change	Autoimmune ILDs	218	4.98 (5.52)	0.59	0.670
	IIPs	286	5.16 (5.15)		
	Sarcoidosis	45	4.51 (5.88)		
	Others ILDs	27	4.30 (3.61)		

For peer review only

	Hypersensitivity pneumonitis	134	5.63 (6.93)		
HR at the beginning of 6MWT	Autoimmune ILDs	219	92.21 (15.09)	2.22	0.065
	IIPs	286	89.13 (13.41)		
	Sarcoidosis	45	88.44 (14.46)		
	Others ILDs	27	91.81 (13.76)		
	Hypersensitivity pneumonitis	135	92.40 (15.61)		
HR at the end of 6MWT	Autoimmune ILDs	277	119.42 (18.71)	1.12	0.344
	IIPs	366	116.46 (17.76)		
	Sarcoidosis	50	118.64 (20.32)		
	Others ILDs	37	119.27 (16.94)		
	Hypersensitivity pneumonitis	176	118.18 (18.63)		
HR zenith	Autoimmune ILDs	219	122.29 (18.61)	1.55	0.187
	IIPs	286	118.88 (18.00)		
	Sarcoidosis	45	124.09 (23.88)		
	Others ILDs	27	122.22 (19.73)		
	Hypersensitivity pneumonitis	135	122.07 (20.53)		
HR change	Autoimmune ILDs	219	-27.23 (14.95)	0.74	0.566
	IIPs	286	-26.77 (13.73)		
	Sarcoidosis	45	-29.89 (14.32)		
	Others ILDs	27	-24.48 (12.92)		
	Hypersensitivity pneumonitis	134	-26.54 (14.52)		

3.4 Correlation between the outcomes of 6MWT and Cardiopulmonary function.

In general, there is a positive correlation between SpO_2 and cardiopulmonary function, whereas heart rate and Borg scale were negatively correlated to and cardiopulmonary function. Specifically, NYHA grade had a strong correlation with the distance of 6MWT (r=0.82, p<0.01). SpO_2 at the end of 3 min had the strongest correlation to patients' FVC (r=0.29, p<0.01), FEV_1 (r=0.24, p<0.01) and DL_{CO} (r=0.41, p<0.01). Details are shown in **Table 4.**

Table 4. Correlation between the outcomes of 6MWT and cardiopulmonary function.

Outcomes of 6MWT	NYHA	FVC	FEV_1	DL_{CO}

For peer review only

6MWD	*r*	**0.82****	0.24**	0.17**	0.26**
	n	751	846	846	806
SpO_2 at the beginning	*r*	0.29**	0.18**	0.17**	0.26**
	n	750	644	644	604
SpO_2 at end of 1 min	*r*	0.27**	0.24**	0.21**	0.31**
	n	748	642	642	602
SpO_2 at end of 2 min	*r*	0.28**	0.27**	.23**	.37**
	n	749	643	643	603
SpO_2 at end of 3 min	*r*	0.29**	**0.29****	**0.24****	**0.41****
	n	749	643	643	603
SpO_2 at end of 4 min	*r*	0.27**	0.27**	0.23**	0.40**
	n	749	643	643	603
SpO_2 at end of 5 min	*r*	0.27**	0.27**	0.23**	0.39**
	n	749	643	643	603
SpO_2 at end of 6 min	*r*	0.26**	0.27**	0.22**	0.37**
	n	748	642	642	602
SpO_2 nadir	*r*	0.27**	0.28**	0.24**	0.38**
	n	750	644	644	604
SpO_2 change	*r*	-0.18**	-0.24**	-0.19**	-0.32**
	n	748	642	642	602
Borg scale at the end	*r*	-0.25**	-0.15**	-0.11**	-0.20**
	n	726	828	828	790
HR at the beginning	*r*	-0.02	-0.18**	-0.21**	-0.10*
	n	750	644	644	604
HR at end of 1 min	*r*	0.16**	-0.08	-0.12**	0.00
	n	749	643	643	603
HR at end of 2 min	*r*	0.18**	-0.08*	-0.11**	-0.02
	n	749	643	643	603
HR at end of 3 min	*r*	0.18**	-0.08	-0.11**	-0.01

For peer review only

	n	749	643	643	603
HR at end of 4 min	*r*	0.19**	-0.06	-0.11**	-0.03
	n	749	643	643	603
HR at end of 5 min	*r*	0.22**	-0.05	-0.09*	-0.02
	n	749	643	643	603
HR at end of 6 min	*r*	0.24**	-0.04	-0.09*	-0.02
	n	749	643	643	603
HR zenith	*r*	0.19**	-0.03	-0.09*	0.01
	n	750	644	644	604
HR change	*r*	-0.32**	-0.14**	-0.11**	-0.09*
	n	749	643	643	603

** means $p< 0.01$, * means $p<0.05$

Discussion

In terms of the features of outcomes of 6MWT in our study, the average of 6MWD in our study, with 457.28 (98.40), was at a moderate level compared to previous studies.[25,26] Since a bigger sample size and a more various ILD sub-group disease were involved, this data is much more representative. HR increases continuously, while SpO_2 generously decreases with a slight upward trend in the fifth minute. However, we have different results compared to Plessis et al..[27] Their data showed a small increase in the fourth minute, but in our study, this small increase was presented in the fifth minute. Additionally, there was no sharp drop of SpO_2 in our study, which appears in their study. Thoracic Society and European Respiratory Society Technical Standard suggest that a 6MWT should be terminated when SpO_2 falls below 80%.[28] SpO_2 less than 88% during exercise is considered as desaturation significantly and be recommended to given oxygen supplement.[29,30,31] From our results, SpO_2 nadir of 226 patients (30.1%) were lower than 88. Moreover, 70 patients (9.3%) whose SpO_2 less than 80 in this study completed 6MWT without breathless or tired to continue, or chest pain, leg cramps, unsteady gait, diaphoresis, or a pale or ashen appearance. Our findings indicated that it's unwarranted to stop 6MWT when ILD patients only have a desaturation without other termination indications, which was in line with Afzal et al..[32] Since SpO_2 nadir has always been considered as an important outcome of 6MWT, our research revealed that 63.87% and 78.27% participants' SpO_2 nadir appeared at the end of third and fourth minute respectively. Besides, SpO_2 nadir in the third

For peer review only

and sixth minutes was statistically equal. This finding indicated that the 6-minute walking time is scientific and appropriate. It is also valuable for guiding clinical practice for hospitals that are unable to monitor patients' data continuously. Oxygen titration is generally done with the 6-minute walk test (6MWT) to determine the O_2 flow preventing oxygen saturation by pulse oximetry (SpO_2) from falling below 88%. According to Giovacchini et al.,[33] after a certain dose of oxygen was given, patients' SpO_2 should be higher than 88 and stable for three minutes. Due to the result that nearly 80% of patients' SpO_2 nadir has appeared at the end of the fourth minute, we strongly recommended this duration should be four minutes, not three.

Compared with the limited research, Garin et al. proved that there was no significant difference between IPF and SSc-ILD on 6MWD and dyspnea.[34] However, SpO_2 after 6MWT was significantly higher and the Borg dyspnea score was significantly lower in patients with dermatomyositis than in patients with idiopathic interstitial pneumonia in another research.[35] As is known to all, ILD contains multiple sub-group. Our results further demonstrated that there is no difference in 6MWT outcomes between different sub-types of ILD, including 6MWD, Borg score, and $SpO_{2.}$

Similar to previous studies, 6MWD and SpO_2 had a positive correlation with objective indexes of cardiopulmonary function, such as NYHA, FVC, FEV_1, and DL_{CO},[5,8-10] while Borg score was inversely correlated with the above outcomes.[28] However, patients' heart rate correlates positively and slightly with NYHA, whereas it has a very slight negative association with FVC, FEV_1, and DL_{CO}. Hence, it's clear that patients' SpO_2 and distance are more valuable than heart rate in the prediction of objective indexes of cardiopulmonary function and subjective degree of dyspnea. What's more, we identified a noticeable difference with the outcome from Nishiyama *et al.*.[25] For FVC and DL_{CO}, SpO_2 has stronger correlations with them than 6MWD. Most research believed that lower FVC and DL_{CO} were associated with poor prognosis and high mortality.[16,36-38] However, it can be argued that DL_{CO} levels are better suited to this goal compared to FVC, capturing the combined impact on the pulmonary reserve of IPF, emphysema, and pulmonary hypertension.[39] The strong correlation between DL_{CO} and SpO_2 indicated that clinical practitioners should focus on the SpO_2 of patients when interpreting the results of 6MWT in patients with ILD. Nevertheless, previous studies have not made a comprehensive comparison on the most valuable time of the patient's SpO_2. A previous study focuses on the correlation between SpO_2 nadir, SpO_2 change, and pulmonary function, and it revealed that SpO_2 nadir and SpO_2 change had the same degree of correlation with FVC, FEV_1,

and DL_{CO}.[40] Although the previous study has highlighted the importance predictive value of SpO_2 nadir,[41] our findings illustrated that the SpO_2 at the end of the third minute may be more valuable than above two, which was recommended that SpO_2 at the end of the third minute could be regarded as an outcome anchor in the verification of the effect of a lung rehabilitation program or other intervention research. At least, compared with 6MWD, this index is more objective and less affected by non-disease factors, such as age, gender, shorter corridor, and inappropriate walking shoes.[42-44] Hence, the third-minute SpO_2 was recommended as a valuable indicator for the evaluation of lung function.

5. Limitations and future research directions

Although this study firstly employs wearable monitors to obtain detailed outcomes throughout the 6MWT and firstly compares outcomes between different sub-type of ILD diseases, several limitations are taken into account when interpreting the findings. Firstly, although we have deduced that the SpO_2 has a strong value in predicting patients' prognosis other than other outcomes of 6MWT, we did not follow up on the outcomes related to prognosis and mortality by longitudinal study. Future research can focus on exploring the association between the outcomes of 6MWT at the key point and prognosis. Secondly, we haven't recorded the HR, SpO_2 one minute after the end of the test as other studies recommended, which may affect the integral understanding of the HR and SpO_2 tendency throughout the 6MWT. And it was expected to be supplemented in the future.

6. Conclusions

Notwithstanding the above limitations, this study demonstrated the tendency of the HR and SpO_2 from the beginning to the end of 6MWT. A nearly 10% of patients whose SpO_2 was less than 80% completed 6MWT without any discomfort indicated that it's unwarranted to stop 6MWT when ILD patients only have a desaturation without other termination indications. Nearly 80% of patients' SpO_2 nadir appears at the end of the fourth minute, which provided evidence for oxygen titration that it's better to maintain SpO_2 above 88% for four minutes. Besides, when a pulmonary function test is unfeasible, the third-minute SpO_2 of 6MWT can be an alternative to predict patients' prognosis. Although there was no significant difference between sub-groups of ILD in 6MWD, which proves that the findings summarized by such a large sample size can be implemented in all kinds of ILD.

Acknowledgments

The authors thank the other medical works for facilitating our data collection, they are Yanni

For peer review only

XU, Yanfen PAN, Yanqiu LV and Xian LI.

Authors' contributions

LJ and WX were involved in the design of the study. SM, LX, and WH were responsible for the data collection. LX was involved in data analysis. LJ drafted the manuscript. All the authors made contributions to the revision and approved the final version.

Funding

This research was supported by the First Affiliated Hospital of Guangzhou Medical University, and the funding number is ZH201822.

Ethics approval

Ethical principles of the Declaration of Helsinki was utilized for guide this study, and the research protocol has been approved by the ethics committee of the First Affiliated Hospital of Guangzhou Medical University. The approval number is 2020 No.K-45.

Competing interests

The authors declare no competing interests.

Data sharing statement

Data were avaliable upon resonable request from Xiaobing Wu (wuxiaobing_gz@163.com).

Reference

1 Flaherty K R , Brown K K , Wells A U , et al. Design of the PF-ILD trial: a double-blind, randomised, placebo-controlled phase III trial of nintedanib in patients with progressive fibrosing interstitial lung disease. BMJ Open Respiratory Research, 2017, 4(1). https://doi.org/10.1136/bmjresp-2017-000212

2 Mikolasch, T. A., Porter, J. C. Transbronchial cryobiopsy in the diagnosis of interstitial lung disease: a cool new approach. Respirology (Carlton, Vic.), 2014, 19(5), 623-624. https://doi.org/10.1111/resp.12320

3 Swigris, J. J., Stewart, A. L., Gould, M. K., et al. Patients' perspectives on how idiopathic pulmonary fibrosis affects the quality of their lives. Health and quality of life outcomes, 2005, 3(1), 61. https://doi.org/10.1186/1477-7525-3-61

4 ATS Committee on Proficiency Standards for Clinical Pulmonary Function Laboratories. ATS statement: guidelines for the six-minute walk test. Am J Respir Crit Care Med, 2002, 166, 111-117. https://doi.org/10.4067/S0717-73482009000100003

For peer review only

5 Caminati, A., Bianchi, A., Cassandro, R., et al. Walking distance on 6-MWT is a prognostic factor in idiopathic pulmonary fibrosis. Respiratory medicine, 2009, 103(1), 117-123. https://doi.org/10.1016/j.rmed.2008.07.022

6 Lancaster, L. H. Utility of the six-minute walk test in patients with idiopathic pulmonary fibrosis. Multidisciplinary respiratory medicine, 2018, 13(1), 1-7. https://doi.org/10.1186/s40248-018-0158-z

7 Wallaert, B., Monge, E., Le Rouzic, O., et al. Physical activity in daily life of patients with fibrotic idiopathic interstitial pneumonia. Chest, 2013, 144(5), 1652-1658. https://doi.org/10.1378/chest.13-0806

8 Bois, R. M. D., Weycker, D., Albera, C., et al. Six-minute-walk test in idiopathic pulmonary fibrosis: test validation and minimal clinically important difference. American Journal of Respiratory and Critical Care Medicine, 2011, 183(9), 1231-1237. https://doi.org/10.1164/rccm.201007-1179OC

9 Nathan, S. D., Du Bois, R. M., Albera, C., et al. Validation of test performance characteristics and minimal clinically important difference of the 6-minute walk test in patients with idiopathic pulmonary fibrosis. Respiratory medicine, 2015, 109(7), 914-922. https://doi.org/10.1016/j.rmed.2015.04.008

10 Mura, M., Ferretti, A., Ferro, O.,et al. Functional predictors of exertional dyspnea, 6-min walking distance and HRCT fibrosis score in idiopathic pulmonary fibrosis. Respiration, 2006, 73(4), 495-502. https://doi.org/10.1159/000089656

11 Kozu, R., Jenkins, S., Senjyu, H. Evaluation of activity limitation in patients with idiopathic pulmonary fibrosis grouped according to Medical Research Council dyspnea grade. Archives of physical medicine and rehabilitation, 2014, 95(5), 950-955. https://doi.org/10.1016/j.apmr.2014.01.016

12 Manali, E. D., Lyberopoulos, P., Triantafillidou, C., et al. MRC chronic Dyspnea Scale: Relationships with cardiopulmonary exercise testing and 6-minute walk test in idiopathic pulmonary fibrosis patients: a prospective study. BMC pulmonary medicine, 2010, 10(1), 32. https://doi.org/10.1186/1471-2466-10-32

13 Eaton, T., Young, P., Milne, D., et al. Six-minute walk, maximal exercise tests: reproducibility in fibrotic interstitial pneumonia. American journal of respiratory and critical care medicine, 2005,

For peer review only

171(10), 1150-1157. https://doi.org/10.1164/rccm.200405-578OC

14 Lama, V. N., Flaherty, K. R., Toews, G. B., et al. Prognostic value of desaturation during a 6-minute walk test in idiopathic interstitial pneumonia. American journal of respiratory and critical care medicine, 2003, 168(9), 1084-1090. https://doi.org/10.1164/rccm.200302-219OC

15 Przybyowski T , Tomalak W , Siergiejko Z , et al. Polish Respiratory Society guidelines for the methodology and interpretation of the 6 minute walk test (6MWT). Pneumonologia i alergologia polska, 2015, 83(4):283-297.doi:10.5603/PiAP.2015.0048

16 Brown, A. W., Nathan, S. D. The value and application of the 6-minute-walk test in idiopathic pulmonary fibrosis. Annals of the American Thoracic Society, 2018, 15(1), 3-10. https://doi.org/10.1513/AnnalsATS.201703-244FR

17 Holland, A. E., Spruit, M. A., Troosters, T., et al. An official European Respiratory Society/American Thoracic Society technical standard: field walking tests in chronic respiratory disease. Eur Respir J, 2014, 44(6), 1428-46. https://doi.org/doi:10.1183/09031936.00150314

18 Borg G A, Psychophysical bases of perceived exertion .Med Sci Sports Exerc, 1982, 14: 377-81.https://doi.org/10.1249/00005768-198205000-00012

19 Williams, N. The Borg rating of perceived exertion (RPE) scale. Occupational Medicine, 2017, 67(5), 404-405. https://doi.org/10.1093/occmed/kqx063

20 Miller-Davis, C., Marden, S., Leidy, N. K. The New York Heart Association Classes and functional status: what are we really measuring?. Heart & Lung, 2006, 35(4), 217-224. https://doi.org/10.1016/j.hrtlng.2006.01.003

21 Harvey, Réjane M. Nomenclature and Criteria for Diagnosis of Diseases of the Heart and Blood Vessels. Journal of the American Medical Association, 1994, 153(9):2054-2054.

22 Enright, P. L.. The six-minute walk test. Respiratory care, 2003, 48(8), 783-785. https://doi.org/10.1093/eurheartj/ehi452

23 Hubbard, R., Johnston, I., Britton, J. Survival in patients with cryptogenic fibrosing alveolitis: a population-based cohort study. Chest, 1998, 113(2), 396-400. https://doi.org/10.1378/chest.113.2.396

24 Cottin, V., Hirani, N. A., Hotchkin, D. L.,et al. Presentation, diagnosis and clinical course of the spectrum of progressive-fibrosing interstitial lung diseases. European Respiratory Review, 2018, 27(150). https://doi.org/10.1183/16000617.0076-2018

For peer review only

25 Nishiyama, O., Yamazaki, R., Sano, H., et al. Pulmonary hemodynamics and six-minute walk test outcomes in patients with interstitial lung disease. Canadian respiratory journal, 2016. https://doi.org/10.1155/2016/3837182

26 Chetta, A., Aiello, M., Foresi, A., et al. Relationship between outcome measures of six-minute walk test and baseline lung function in patients with interstitial lung disease. Sarcoidosis, Vasculitis, and Diffuse Lung Diseases: Official Journal of WASOG, 2001, 18(2), 170-175. https://doi.org/10.1053/rmed.2001.1087

27 Plessis J , Fernandes S , Jamal R , et al. Exertional hypoxemia is more severe in fibrotic interstitial lung disease than in COPD[J]. Respirology, 2017.

28 Holland AE, Spruit MA, Troosters T, et al. An offificial European Respiratory Society/American Thoracic Society technical standard: field walking tests in chronic respiratory disease. Eur Respir J 2014; 44:1428-46

29 Casanova C, Cote C, Marin JM et al. Distance and oxygen desaturation during the 6-min walk test as predictors of long-term mortality in patients with COPD. Chest 2008; 134: 746−752. doi: 10.1378/chest.08-0520.

30 Salzman SH. The 6-min walk test: clinical and research role, technique, coding, and reimbursement. Chest 2009;135(5): 1345e52.

31 Hook JL, Arcasoy SM, Zemmel D, Bartels MN, Kawut SM, Lederer DJ. Titrated oxygen requirement and prognostication in idiopathic pulmonary fifibrosis. Eur Respir J 2012;39(2): 359e65.

32 Afzal S , Burge A T , Lee A L , et al. Should the 6-Minute Walk Test Be Stopped If Oxyhemoglobin Saturation Falls Below 80%?[J]. Archives of Physical Medicine & Rehabilitation, 2018.

33 Giovacchini CX, Mathews AM, Lawlor BR, MacIntyre NR. Titrating Oxygen Requirements During Exercise: Evaluation of a Standardized Single Walk Test Protocol. Chest. 2018 Apr;153(4):922-928. doi: 10.1016/j.chest.2017.11.009.

34 Garin MC, Highland KB, Silver RM, Strange C. Limitations to the 6-minute walk test in interstitial lung disease and pulmonary hypertension in scleroderma. J Rheumatol. 2009 Feb;36(2):330-6. doi: 10.3899/jrheum.080447. Epub 2009 Jan 22.

35 Someya F, Mugii N. Limitations to the 6-minute walk test in dermatomyositis with interstitial

For peer review only

lung disease in comparison with idiopathic interstitial pneumonia. Clin Med Insights Circ Respir Pulm Med. 2013;7:1-6. doi: 10.4137/CCRPM.S10764.

36 Holland, A. E., Dowman, L., Fiore, J.,et al. Cardiorespiratory responses to 6-minute walk test in interstitial lung disease: not always a submaximal test. BMC pulmonary medicine, 2014, 14(1), 136. https://doi.org/10.1186/1471-2466-14-136

37 Ostoji P , Damjanov N . Different clinical features in patients with limited and diffuse cutaneous systemic sclerosis. Clinical Rheumatology, 2006, 25(4):453-457. https://doi.org/10.1007/s10067-005-0041-0

38. Khanna Dinesh,Tashkin Donald P,Denton Christopher P et al. Etiology, Risk Factors, and Biomarkers in Systemic Sclerosis with Interstitial Lung Disease. Am J Respir Crit Care Med, 2020, 201: 650-660. https://doi.org/10.1164/rccm.201903-0563CI

39 Wells A U . Novel exploratory data in interstitial lung disease[J]. Respirology, 2019, 24(8).

40 Du Plessis JP, Fernandes S, Jamal R, Camp P, Johannson K, Schaeffer M, Wilcox PG, Guenette JA, Ryerson CJ. Exertional hypoxemia is more severe in fibrotic interstitial lung disease than in COPD. Respirology. 2018 Apr;23(4):392-398. doi: 10.1111/resp.13226.

41 Lettieri, C. J. , Nathan, S. D. , Browning, R. F. ,et al. The distance-saturation product predicts mortality in idiopathic pulmonary fibrosis. Respiratory Medicine, 2006, 100(10), 1734-1741. https://doi.org/10.1016/j.rmed.2006.02.004

42 Nishiyama O, Yamazaki R, Sano H, Iwanaga T, Higashimoto Y, Kume H, Tohda Y. Pulmonary Hemodynamics and Six-Minute Walk Test Outcomes in Patients with Interstitial Lung Disease. Can Respir J. 2016;2016:3837182. doi: 10.1155/2016/3837182. Epub 2016 May 18. PMID: 27445533; PMCID: PMC4904526.

43 Beekman E, Mesters I, Hendriks E.J et al. Course length of 30 metres versus 10 metres has a significant influence on Six-minute walk distance in patients with COPD: an experimental crossover study. J Physiother 2013; 59: 169−176. doi: 10.1016/S1836-9553(13)70181-4.

44 Barrios JA, Crenshaw JR, Royer TD, Davis IS. Walking shoes and laterally wedged orthoses in the clinical management of medial tibiofemoral osteoarthritis: a one-year prospective controlled trial. Knee 2009; 16: 136-42. doi: 10.1016/j. knee.2008.10.002.

Figure 1. Description of SpO_2 and HR in patients with ILD during 6MWT

For peer review only

Figure 2. Statistical analysis chart of SpO_2 in ILD patients within six minutes

For peer review only

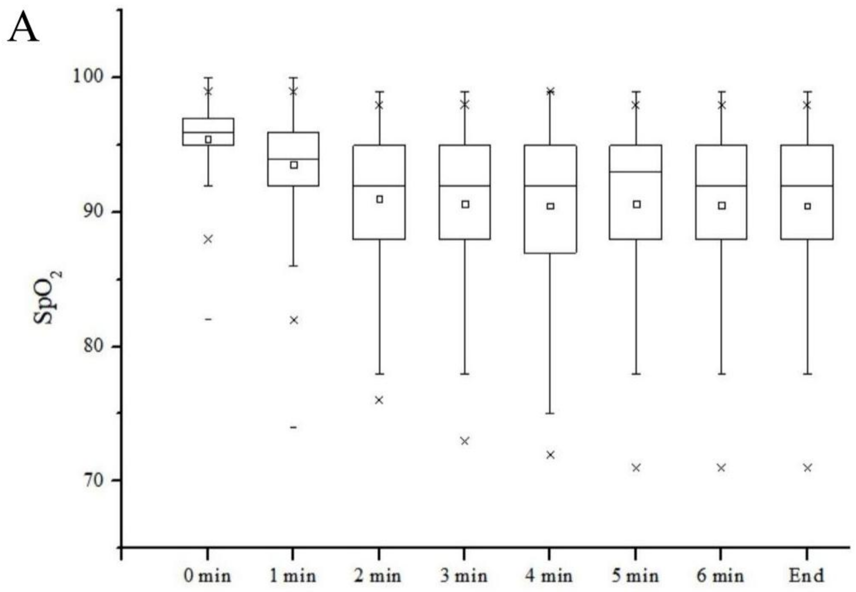
A
SpO2
100
90
80
70
0 min
1 min
2 min
3 min
4 min
5 min
6 min
End

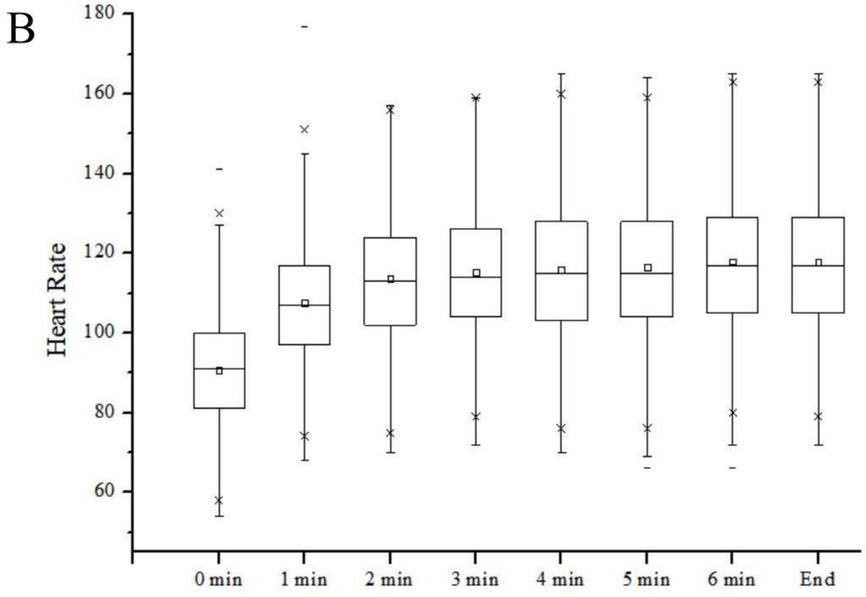
B
Heart Rate
180
160
140
120
100
80
60
0 min
1 min
2 min
3 min
4 min
5 min
6 min
End

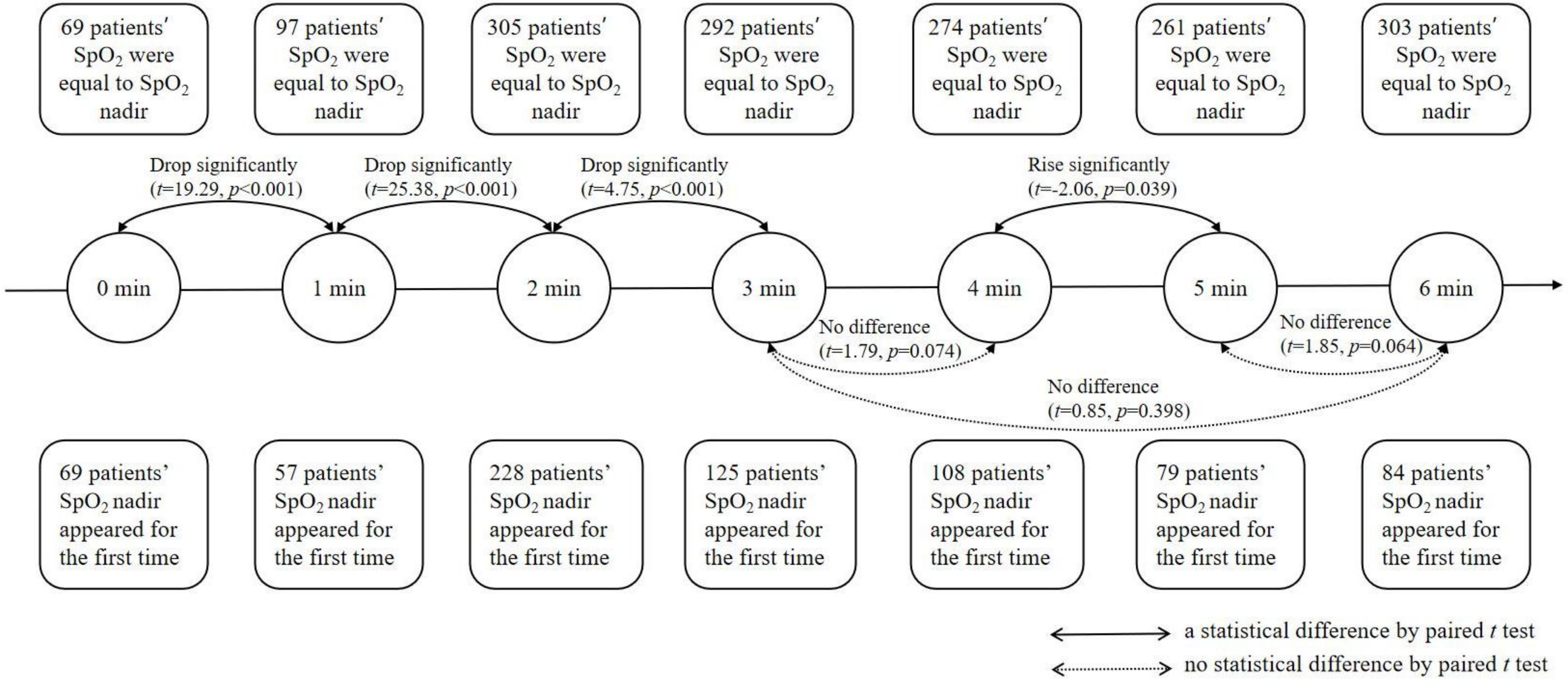
69 patients' SpO2 were equal to SpO2 nadir
97 patients' SpO2 were equal to SpO2 nadir
305 patients' SpO2 were equal to SpO2 nadir
292 patients' SpO2 were equal to SpO2 nadir
274 patients' SpO2 were equal to SpO2 nadir
261 patients' SpO2 were equal to SpO2 nadir
303 patients' SpO2 were equal to SpO2 nadir
Drop significantly (t=19.29, p<0.001)
Drop significantly (t=25.38, p<0.001)
Drop significantly (t=4.75, p<0.001)
Rise significantly (t=-2.06, p=0.039)
0 min
1 min
2 min
3 min
4 min
5 min
6 min
No difference (t=1.79, p=0.074)
No difference (t=1.85, p=0.064)
No difference (t=0.85, p=0.398)
69 patients' SpO2 nadir appeared for the first time
57 patients' SpO2 nadir appeared for the first time
228 patients' SpO2 nadir appeared for the first time
125 patients' SpO2 nadir appeared for the first time
108 patients' SpO2 nadir appeared for the first time
79 patients' SpO2 nadir appeared for the first time
84 patients' SpO2 nadir appeared for the first time
a statistical difference by paired t test
no statistical difference by paired t test

STROBE Statement—checklist of items that should be included in reports of observational studies

	Item No	Recommendation	Page No
**Title and abstract**	1	(*a*) Indicate the study's design with a commonly used term in the title or the abstract	1
		(*b*) Provide in the abstract an informative and balanced summary of what was done and what was found	1
**Introduction**			
Background/rationale	2	Explain the scientific background and rationale for the investigation being reported	1-2
Objectives	3	State specific objectives, including any prespecified hypotheses	2
**Methods**			
Study design	4	Present key elements of study design early in the paper	3
Setting	5	Describe the setting, locations, and relevant dates, including periods of recruitment, exposure, follow-up, and data collection	3
Participants	6	(*a*) *Cohort study*—Give the eligibility criteria, and the sources and methods of selection of participants. Describe methods of follow-up<br>*Case-control study*—Give the eligibility criteria, and the sources and methods of case ascertainment and control selection. Give the rationale for the choice of cases and controls<br>*Cross-sectional study*—Give the eligibility criteria, and the sources and methods of selection of participants	3
		(*b*) *Cohort study*—For matched studies, give matching criteria and number of exposed and unexposed<br>*Case-control study*—For matched studies, give matching criteria and the number of controls per case	-
Variables	7	Clearly define all outcomes, exposures, predictors, potential confounders, and effect modifiers. Give diagnostic criteria, if applicable	3-4
Data sources/ measurement	8*	For each variable of interest, give sources of data and details of methods of assessment (measurement). Describe comparability of assessment methods if there is more than one group	*4*
Bias	9	Describe any efforts to address potential sources of bias	4
Study size	10	Explain how the study size was arrived at	3
Quantitative variables	11	Explain how quantitative variables were handled in the analyses. If applicable, describe which groupings were chosen and why	4
Statistical methods	12	(*a*) Describe all statistical methods, including those used to control for confounding	4
		(*b*) Describe any methods used to examine subgroups and interactions	4
		(*c*) Explain how missing data were addressed	4
		(*d*) *Cohort study*—If applicable, explain how loss to follow-up was addressed<br>*Case-control study*—If applicable, explain how matching of cases and controls was addressed<br>*Cross-sectional study*—If applicable, describe analytical methods taking account of sampling strategy	-
		(*e*) Describe any sensitivity analyses	4

Continued on next page

For peer review only - http://bmjopen.bmj.com/site/about/guidelines.xhtml

**Results**			
Participants	13*	(a) Report numbers of individuals at each stage of study—eg numbers potentially eligible, examined for eligibility, confirmed eligible, included in the study, completing follow-up, and analysed	5
		(b) Give reasons for non-participation at each stage	5
		(c) Consider use of a flow diagram	5
Descriptive data	14*	(a) Give characteristics of study participants (eg demographic, clinical, social) and information on exposures and potential confounders	5
		(b) Indicate number of participants with missing data for each variable of interest	5
		(c) *Cohort study*—Summarise follow-up time (eg, average and total amount)	5
Outcome data	15*	*Cohort study*—Report numbers of outcome events or summary measures over time	-
		Case-control study—Report numbers in each exposure category, or summary measures of exposure	-
		Cross-sectional study—Report numbers of outcome events or summary measures	*5*
Main results	16	(*a*) Give unadjusted estimates and, if applicable, confounder-adjusted estimates and their precision (eg, 95% confidence interval). Make clear which confounders were adjusted for and why they were included	5
		(*b*) Report category boundaries when continuous variables were categorized	5
		(*c*) If relevant, consider translating estimates of relative risk into absolute risk for a meaningful time period	-
Other analyses	17	Report other analyses done—eg analyses of subgroups and interactions, and sensitivity analyses	5
**Discussion**			
Key results	18	Summarise key results with reference to study objectives	6-7
Limitations	19	Discuss limitations of the study, taking into account sources of potential bias or imprecision. Discuss both direction and magnitude of any potential bias	6-7
Interpretation	20	Give a cautious overall interpretation of results considering objectives, limitations, multiplicity of analyses, results from similar studies, and other relevant evidence	6-7
Generalisability	21	Discuss the generalisability (external validity) of the study results	6-7
**Other information**			
Funding	22	Give the source of funding and the role of the funders for the present study and, if applicable, for the original study on which the present article is based	8

*Give information separately for cases and controls in case-control studies and, if applicable, for exposed and unexposed groups in cohort and cross-sectional studies.

Note: An Explanation and Elaboration article discusses each checklist item and gives methodological background and published examples of transparent reporting. The STROBE checklist is best used in conjunction with this article (freely available on the Web sites of PLoS Medicine at http://www.plosmedicine.org/, Annals of Internal Medicine at http://www.annals.org/, and Epidemiology at http://www.epidem.com/). Information on the STROBE Initiative is available at www.strobe-statement.org.

For peer review only

BMJ Open

Features and Predictive Value of Six-minute Walk Test Outcomes in Interstitial Lung Disease: An Observation Study using Wearable Monitors

Journal:	*BMJ Open*
Manuscript ID	bmjopen-2021-055077.R1
Article Type:	Original research
Date Submitted by the Author:	21-Mar-2022

Complete List of Authors:	LI, Jiaying; The University of Hong Kong, School of Nursing<br>LI, Xiaoyan; First Affiliated Hospital of Guangzhou Medical College, Guangzhou Institute of Respiratory Health<br>DENG, Miaozhen; First Affiliated Hospital of Guangzhou Medical College, Guangzhou Institute of Respiratory Health<br>LIANG, Xinyin; First Affiliated Hospital of Guangzhou Medical College, Guangzhou Institute of Respiratory health<br>WEI, Huiqun; First Affiliated Hospital of Guangzhou Medical College, Guangzhou Institute of Respiratory Health<br>Wu, Xiaobing; First Affiliated Hospital of Guangzhou Medical College, Department of Internal Medicine

**Primary Subject Heading**:	Respiratory medicine
Secondary Subject Heading:	Respiratory medicine
Keywords:	Interstitial lung disease < THORACIC MEDICINE, Rehabilitation medicine < INTERNAL MEDICINE, RESPIRATORY MEDICINE (see Thoracic Medicine)

SCHOLARONE™
Manuscripts

I, the Submitting Author has the right to grant and does grant on behalf of all authors of the Work (as defined in the below author licence), an exclusive licence and/or a non-exclusive licence for contributions from authors who are: i) UK Crown employees; ii) where BMJ has agreed a CC-BY licence shall apply, and/or iii) in accordance with the terms applicable for US Federal Government officers or employees acting as part of their official duties; on a worldwide, perpetual, irrevocable, royalty-free basis to BMJ Publishing Group Ltd ("BMJ") its licensees and where the relevant Journal is co-owned by BMJ to the co-owners of the Journal, to publish the Work in this journal and any other BMJ products and to exploit all rights, as set out in our licence.

The Submitting Author accepts and understands that any supply made under these terms is made by BMJ to the Submitting Author unless you are acting as an employee on behalf of your employer or a postgraduate student of an affiliated institution which is paying any applicable article publishing charge ("APC") for Open Access articles. Where the Submitting Author wishes to make the Work available on an Open Access basis (and intends to pay the relevant APC), the terms of reuse of such Open Access shall be governed by a Creative Commons licence – details of these licences and which Creative Commons licence will apply to this Work are set out in our licence referred to above.

Other than as permitted in any relevant BMJ Author's Self Archiving Policies, I confirm this Work has not been accepted for publication elsewhere, is not being considered for publication elsewhere and does not duplicate material already published. I confirm all authors consent to publication of this Work and authorise the granting of this licence.

For peer review only

Title Page

Author and Co-author's name

Jiaying LI[1], Xiaoyan LI[2], Miaozhen DENG[2], Xinyin LIANG[2], Huiqun WEI[2], Xiaobing WU[2]

Information of corresponding author

Xiaobing WU, First Affiliated Hospital of Guangzhou Medical College. No.151 Yanjiang West Road, Yuexiu District, Guangzhou, Guangdong, China, wuxiaobing_gz@163.com, +86 13711663164

Information of co-authors

Jiaying LI, The University of Hong Kong, School of Nursing, Hong Kong, China

Xiaoyan LI, First Affiliated Hospital of Guangzhou Medical College, Guangzhou, China

Miaozhen DENG: First Affiliated Hospital of Guangzhou Medical College, Guangzhou, China

Xinyin LIANG: First Affiliated Hospital of Guangzhou Medical College, Guangzhou, China

Huiqun WEI: First Affiliated Hospital of Guangzhou Medical College, Guangzhou, China

Keywords: 6MWT, interstitial lung disease, predictive value, cardiopulmonary function

Word count: 3302

For peer review only

Features and Predictive Value of Six-minute Walk Test Outcomes in Interstitial Lung Disease: An Observation Study using Wearable Monitors

Abstract

Objectives: To describe six-minute walk test (6MWT) outcomes, and to investigate their correlations with cardiopulmonary and lung function among patients with interstitial lung disease (ILD) which was not limited to idiopathic pulmonary fibrosis (IPF).

Methods: We collected patients' demographic data and obtained minute-by-minute 6MWT outcomes. Modified Borg scale was employed to assess patients' dyspnea, whereas New York Heart Association (NYHA) classification and pulmonary function test were utilized to evaluate patients' cardiopulmonary functions.

Results: Heart rate (HR) exhibited a continuous upward trend, while SpO_2 exhibited an overall downward with a slight increase at the fifth minute. The SpO_2 nadir for 70 patients (9.3%) was lower than 80%. Further, the SpO_2 nadir for 78.27% of the participants appeared at the end of the fourth minute. The six-minute walk distance (6MWD) had the strongest correlation with NYHA classification (r=0.82, p < 0.01). The ratio of 6MWD and predicted 6MWD was most correlated to forced expiratory volume in the first second (FEV_1) (r=0.30, p < 0.01) and forced vital capacity (FVC) (r=0.30, p < 0.01). SpO_2 at 3 min had the strongest correlation to patients' diffusing capacity of the lungs for carbon monoxide (DL_{CO}) (r=0.41, p < 0.01). We found significant differences in 6MWD (F = 2.44, p = 0.033), SpO_2 change (F = 2.58, p = 0.025), HR at 0 min (F = 2.87, p = 0.014), HR at end of 6 min (F = 2.58, p = 0.025), and HR zenith (F = 2.64, p = 0.022) between the sub-types of ILD.

Conclusion: This observation provided an important evidence regarding oxygen titration. It is better to maintain SpO_2 above 88% for four minutes instead of three minutes. SpO_2 at the third minute was the most valuable predictor of patients' lung function. 6MWD and SpO_2 changes were more discriminative in sub-types.

Keywords: 6MWT, interstitial lung disease, predictive value, cardiopulmonary function, sub-types

Strengths of this study

- The study included a large sample size of ILD patients not limited to IPF.

For peer review only

- This was the first study to compare 6MWT outcomes between multiple sub-types of ILD.
- This study described the tendency of heart rate and SpO_2 minute-by-minute during 6MWT.

Limitations of this study

- Lack of follow-up hindered the prediction of patients' long-term clinical outcomes using 6MWT.

Background

Interstitial lung disease (ILD) is a group of more than 200 kinds of diseases characterized by pulmonary inflammation, accompanied with or without fibrosis.[1,2] Patients diagnosed with ILD mostly have dyspnea and decreased tolerance to exercise.[3] The six-minute walk test (6MWT) is widely used to assess patients' performance ability with different cardiopulmonary-related diseases, which provides essential outcomes that cannot be obtained otherwise by standardized pulmonary function testing.[4]

Till date, most 6MWT-related studies have focused on idiopathic pulmonary fibrosis (IPF), which is the most common type of ILD. Previous studies have investigated the outcomes of 6MWT, most of which centered on the six-minute walk distance (6MWD) and percutaneous oxygen saturation (SpO_2). However, 6MWT is ideal in predicting patients' clinical outcomes. Previous studies found that 6MWD and oxygen desaturation are associated with mortality in patients with IPF.[5,6] According to a previous study, 6MWD was an independent positive factor for the physical activity of patients with IPF.[7] In addition, 6MWD had a positive association with the subjective health-related quality of life (HRQL) and objective lung function index,[8-9] which included the predicted percentage of forced vital capacity (FVC) and predicted lung diffusing capacity for carbon monoxide (DLco)[5,8,9] as well as forced expiratory volume in the first second (FEV_{1}),[10] which is a negative predictor for dyspnea.[8,9,11,12] Furthermore, the occurrence of desaturation and changes in SpO_2 during the test were indicators of patients' mortality with IPF.[13,14]

6MWT has multiple associated outcomes that are not restricted to 6MWD and SpO_2. It comprises of the distance walked, heart rate, blood pressure, SpO_2, and dyspnea, as assessed by the Borg scale.[15] Although the predictive value of oxygen desaturation for the clinical outcomes of the patients has been confirmed,[5,6] the most predictive time point of this outcome within the six minutes remains unclear. In

For peer review only

addition, the effect of different sub-types of ILD on 6MWT outcomes have not been evaluated yet. Several studies highlighted the importance of finding the most prognostic outcome of 6MWT,[6,16] and measuring SpO_2 for the entire six minute duration of 6MWT is recommended by the 2014 technical standards of ERS/ATS.[17] In the current study, wearable monitors were used to obtain the precise minute-by-minute data of 6MWT, which facilitated the descriptions and comparisons in detail. The comparison between subgroups of ILD will provide new insights into the distinguishing value of 6MWT outcomes.

Hence, to provide detailed features, the predictive value of 6MWT for cardiopulmonary functions, and its distinguishing value for the sub-types of ILD in the current study, we aimed to: (1) Describe the detailed outcomes of 6MWT outcomes, including the heart rate (HR), SpO_2, blood pressure, Borg score, and walking distance. (2) Identify the correlations between 6MWT outcomes and patients' cardiopulmonary functions. (3) Investigate the effect of the differences between sub-classifications of ILD on 6MWT outcomes.

Methods

Design

This was an observational study using a wearable monitor.

Patients

All the patients were recruited from July 2019 to August 2020 at the Guangzhou Respiratory Health Institute, the biggest respiratory center in China. We identified eligible participants based on the following inclusion and exclusion criteria—we included patients who were diagnosed with interstitial lung disease, or whose condition was feasible to conduct 6MWT. The expert pulmonologist established the diagnosis based on patients' symptoms, the radiologist's opinion from the imaging tests, blood tests results, lung function tests, bronchoscopy, and biopsy. We excluded patients who had walking limitations, including joint restrictions or other critical diseases; and those who experienced myocardial infarction in the previous five days, unstable angina, syncope, symptomatic arrhythmia, severe aortic stenosis, or decompensated heart failure due to another unstable medical issues.[17] After the initial screening, we obtained informed consent from eligible participants before including them in

For peer review only

the study.

Measurements

Demographics questionnaire

The self-designed demographic questionnaire included questions about the age, height, weight, BMI, and sex of the participants.

NYHA functional classification

The New York Heart Association (NYHA) classification was considered as a critical criterion for a comprehensive cardiac diagnosis.[18] It classifies patients into four categories, based on their limitations during physical activity, which ranges from no symptoms with ordinary physical activity (Class I) to symptoms at rest and increased discomfort with any physical activity (Class IV).[19]

Borg scale

The Borg Rating of Perceived Exertion scale was developed by Borg,[20] which is widely used to measure patients' effort and exertion, breathlessness, and fatigue during physical work. A higher score indicated a more severe level of exertion.[21]

Outcomes of 6MWT

According to Enright's recommendations,[22] the primary outcome in our study was 6MWD. We calculated the predicted 6MWD based on equations developed by Enright and Sherrill.[23] For men, the predicted 6MWD = (7.57 * $height_{cm}$) - (5.02 * age) - (1.76 * $weight_{kg}$) - 309m. For women, the predicted 6MWD = (2.11 * $height_{cm}$) - (2.29 * age) - (5.78 * $weight_{kg}$) + 667m. Secondary outcomes include fatigue and dyspnea, arterial oxygen saturation, heart rate, and blood pressure. We measured fatigue and dyspnea by modified Borg scale before and after 6MWT, and used wearable monitors to record patients' arterial oxygen saturation and heart rate during 6MWT. We also recorded the patients' blood pressure before and after the test, and calculate the mean arterial pressure. In this study, 6MWT was conducted without oxygen therapy support. Most of participants had received a 6MWT at the outpatient clinic before their hospital admission. In this case, the learning effect that improves the distance of second walk will be weak.[24] Therefore, we conducted one 6MWT for each patient.

For peer review only

Pulmonary function test

Restriction of lung volumes and dysfunction of diffusion are the main functional respiratory abnormalities. An increased FEV_1/FVC ratio, accompanied by a low total lung capacity, indicates restriction of lung volumes. Previous studies have proven that reduction in Forced Vital Capacity (FVC) and Diffusion Capacity for Carbon Monoxide of the Lung (DL_{CO}) are associated with poor survival rates and prognosis.[25] Therefore, in this study, FVC, forced expiratory volume in one second (FEV1), and DL_{CO} were used for the respiratory function assessment.

Sub-types of ILD

Since ILD encompasses more than 200 parenchymal pulmonary disorders, we divided all the cases into sub-types, to facilitate the analysis. According to the classifications of Cottin *et al.*,[26] the sub-types contains idiopathic interstitial pneumonias, autoimmune ILDs, hypersensitivity pneumonitis, sarcoidosis, and other ILDs. Beause IPF is the most widely studied and the most common type of ILD, we classified it as a dependent category to make the comparisons more detailed. Therefore, we included six sub-types in total.

Data collection

We collected patients' demographic data using a self-designed questionnaire, which was administered after 6MWT. The outcomes of the pulmonary function test and the NYHA functional classification were obtained from the patients' medical records. The Borg scale was employed before and after walking. All the 6MWT-related outcomes were automatically collected using physiological parameters transmission management software during the 6 minutes module (Shenzhen zhongruiqi Electronic Technology Co., Ltd). Since the patients at the center completed all the assessments and tests within three days of their admission, the outcomes of pulmonary function test, NYHA functional classification, and 6MWT were obtained within the next three days.

Analysis

We used the statistical package for the social sciences (SPSS) software version 21.0 (IBM Corporation, Armonk, New York, USA) for data analysis. We used descriptive statistics to summarize the participants' demographics, 6MWT outcomes, Borg grades, NYHA functional classification, and

For peer review only

pulmonary function indexes. Specifically, we described continuous variables as mean and standard deviation (SD), and categorical variables as frequency. After performing a check for normality, we used the paired *t*-test to assess the differences in SpO_2 between each end of the minute. Analysis of variance (ANOVA) were used to assess the differences of 6MWT-related outcomes across the sub-types of ILD. We performed the Pearson correlation analysis to identify the correlations between the 6MWT outcomes and other measurements. Statistical significance was set at $p < 0.05$.

Patient and Public Involvement

No patient involved.

Results

Demographics and characteristics of patients

We included 954 patients with ILD from July 2019 to August 2020. The average age of participants was 55.40 (SSD=12.35) years (range, 14–83 years). The sample included 510 (53.50%) male (Table 1).

Table 1. The demographic and characteristic information of ILD patients (n=954)

**Variables**	**Categories**	***N (%) / Mean(SD)***
Height (cm)	-	161.08 (8.00)
Weight (kg)	-	62.60 (10.60
BMI	-	24.07 (3.39)
Subclass of ILD	Autoimmune ILDs	277（29.00）
	IIPs	195（20.40）
	IPF	171 (17.90)
	Sarcoidosis	50（5.20）
	Hypersensitivity pneumonitis	177（18.60）

For peer review only

Others ILDs	37（3.90）
Missing data	47（4.90）

BMI: body mass index; ILD: interstitial lung disease; ILDs: interstitial lung diseases; IIPs: Idiopathic interstitial pneumonias; IPF: idiopathic pulmonary fibrosis.

Features of 6MWT outcomes among ILD patients

For 750 participants with valid data, the SpO_2 nadir was higher than 80%, and for 524 patients (69.9%) the SpO_2 nadir was higher than 88. Other details are shown in **Table 2.** Figure 1 shows patients' SpO_2 and HR during 6MWT. SpO_2 generally showed a downward trend, but increased slightly at the end of the fifth minute, whereas HR exhibited a sharp increase in the first two minutes and reached a peak before becoming steady. Paired t-test found three significant drops in the SpO_2, which occurred at the first minute (t=19.29, $p < 0.001$), the second minute (t=25.38, $p < 0.001$), and the third minute (t=4.75, $p < 0.001$). This was accompanied by a slightly significant rise at the fourth minute (t=-2.06, $p = 0.039$). **Figure 2** depicts the time point when SpO_2 nadir appears at the first time and the occurrence of SpO_2 nadir at each end of a minute over six minutes. The SpO_2 nadir of 63.87% and 78.27% of the. participants' appeared at the end of the third and the fourth minute, respectively.

Table 2. The features of 6MWT outcomes among ILD patients

Items	N	Minimum	Maximum	Mean (SD)
Systolic blood pressure before 6MWT (mmHg)	950	84	188	124.16 (17.26)
Systolic blood pressure after 6MWT (mmHg)	734	88	242	138.46 (23.19)
Diastolic blood pressure before 6MWT (mmHg)	950	50	128	77.64 (11.58)
Diastolic blood pressure after 6MWT (mmHg)	734	49	167	82.45 (13.44)
Mean arterial pressure before 6MWT (mmHg)	950	62.67	148.00	93.15 (12.16)
Mean arterial pressure before 6MWT (mmHg)	734	66.67	182.00	101.12 (14.89)
Heart rate at 0 min (times/min)	750	50	141	90.97 (14.48)

For peer review only

Heart rate at 1 min (times/min)	749	65	177	107.69 (15.25)
Heart rate at 2 min (times/min)	749	70	192	113.66 (16.33)
Heart rate at 3 min (times/min)	749	68	199	115.43 (16.88)
Heart rate at 4 min (times/min)	749	70	201	116.08 (17.65)
Heart rate at 5 min (times/min)	749	66	195	116.49 (17.77)
Heart rate at 6 min (times/min)	749	66	193	117.97 (18.00)
Heart rate zenith (times/min)	750	70	201	121.38(19.18)
Heart rate change (times/min)	749	-102.00	29.00	-27.07(14.45)
SpO_2 at 0 min (%)	750	82	100	95.49 (2.23)
SpO_2 at 1 min (%)	748	74	100	93.61 (3.46)
SpO_2 at 2 min (%)	749	65	99	91.03 (4.99)
SpO_2 at 3 min (%)	749	65	99	90.62 (5.82)
SpO_2 at 4 min (%)	749	58	99	90.49 (6.13)
SpO_2 at 5 min (%)	749	61	99	90.65 (6.23)
SpO_2 at 6 min (%)	748	56	99	90.54 (6.43)
SpO_2 nadir (%)	750	56.00	99.00	89.09(6.44)
SpO_2 change (%)	748	-10.00	33.00	4.96(5.57)
Distance (meter)	953	53	999	457.28 (98.40)
Distance/ predicted distance (meter)	953	12.49	175.1	84.74 (18.54)

Heart rate change and SpO_2 change were the values at the beginning minus the values at the end of six minutes respectively. 6MWT: six-minute walk test; SpO_2: peripheral capillary oxygen saturation

Difference in 6MWT outcomes among sub-group of ILD patients

For peer review only

Significant differences between the sub-types of ILD were found for 6MWD ($F = 2.44$, $p = 0.033$), SpO_2 change ($F = 2.58$, $p = 0.025$), HR at 0 min ($F = 2.87$, $p = 0.014$), HR at end of 6 min ($F = 2.58$, $p = 0.025$), and HR zenith ($F = 2.64$, $p = 0.022$) (**Table 3**).

Table 3. Difference between sub-groups of ILD diseases in 6MWT outcomes

**Measures**	**Categories**	**N**	**Mean(SD)**	***F***	***p***
Borg score before 6MWT	Autoimmune ILDs	267	0.25 (0.54)	1.59	0.160
	IIPs	192	0.18 (0.50)		
	IPF	167	0.28 (0.51)		
	Sarcoidosis	49	0.18 (0.39)		
	Others ILDs	37	0.35 (0.63)		
	Hypersensitivity pneumonitis	174	0.31 (0.59)		
Borg score after 6MWT	Autoimmune ILDs	267	1.25 (1.15)	0.97	0.433
	IIPs	192	1.32 (1.21)		
	IPF	167	1.37 (1.23)		
	Sarcoidosis	49	1.04 (0.98)		
	Others ILDs	36	1.14 (1.17)		
	Hypersensitivity pneumonitis	174	1.38 (1.15)		
6MWD (meter)	Autoimmune ILDs	277	452.69 (96.25)	2.44	0.033
	IIPs	195	466.07 (96.86)		
	IPF	171	440.70 (87.85)		
	Sarcoidosis	50	479.36 (91.25)		
	Others ILDs	37	482.30 (116.70)		

For peer review only

	Hypersensitivity pneumonitis	177	453.48 (107.31)		
6MWD / predicted 6MWD	Autoimmune ILDs	277	0.84 (0.18)	0.84	0.521
	IIPs	195	0.85 (0.19)		
	IPF	171	0.87 (0.18)		
	Sarcoidosis	50	0.87 (0.17)		
	Others ILDs	37	0.84 (0.23)		
	Hypersensitivity pneumonitis	177	0.84 (0.19)		
SpO_2 at the 0 min (%)	Autoimmune ILDs	219	95.75 (1.98)	2.14	0.059
	IIPs	143	95.15 (2.53)		
	IPF	143	95.14 (2.33)		
	Sarcoidosis	45	95.40 (1.76)		
	Others ILDs	27	95.37 (3.12)		
	Hypersensitivity pneumonitis	135	95.70 (2.21)		
SpO_2 at the end of 6 min (%)	Autoimmune ILDs	218	90.78 (6.07)	2.14	0.059
	IIPs	143	91.07 (5.74)		
	IPF	143	88.91 (6.49)		
	Sarcoidosis	45	90.89 (6.41)		
	Others ILDs	27	91.07 (5.86)		
	Hypersensitivity pneumonitis	134	90.07 (7.85)		
SpO_2 nadir (%)	Autoimmune ILDs	219	89.20 (6.01)	1.421	0.215
	IIPs	143	89.60 (5.52)		

For peer review only

	IPF	143	87.86 (6.56)		
	Sarcoidosis	45	88.89 (6.47)		
	Others ILDs	27	90.15 (5.82)		
	Hypersensitivity pneumonitis	135	88.59 (8.08)		
SpO_2 change (%)	Autoimmune ILDs	218	4.98 (5.52)	2.58	0.025
	IIPs	143	4.08 (5.04)		
	IPF	143	6.23 (5.05)		
	Sarcoidosis	45	4.51 (5.88)		
	Others ILDs	27	4.30 (3.61)		
	Hypersensitivity pneumonitis	134	5.63 (6.93)		
HR at the 0 min (times/min)	Autoimmune ILDs	219	92.21 (15.09)	2.87	0.014
	IIPs	143	91.12 (12.75)		
	IPF	143	87.15 (13.80)		
	Sarcoidosis	45	88.44 (14.46)		
	Others ILDs	27	91.81 (13.76)		
	Hypersensitivity pneumonitis	135	92.40 (15.61)		
HR at the end of 6 min (times/min)	Autoimmune ILDs	219	119.45 (17.65)	2.58	0.025
	IIPs	143	118.76 (18.63)		
	IPF	143	113.05 (15.41)		
	Sarcoidosis	45	118.33 (20.99)		
	Others ILDs	27	116.30 (16.22)		

For peer review only

	Hypersensitivity pneumonitis	135	118.57 (18.77)		
HR zenith (times/min)	Autoimmune ILDs	219	122.29 (18.61)	2.64	0.022
	IIPs	143	121.86 (19.58)		
	IPF	143	115.90 (15.79)		
	Sarcoidosis	45	124.09 (23.88)		
	Others ILDs	27	122.22 (19.73)		
	Hypersensitivity pneumonitis	135	122.07 (20.53)		
HR change (times/min)	Autoimmune ILDs	219	-27.23 (14.95)	0.80	0.547
	IIPs	143	-27.64 (15.23)		
	IPF	143	-25.90 (12.03)		
	Sarcoidosis	45	-29.89 (14.32)		
	Others ILDs	27	-24.48 (12.92)		
	Hypersensitivity pneumonitis	134	-26.54 (14.52)		

ILDs: interstitial lung diseases; IIPs: Idiopathic interstitial pneumonias; IPF: idiopathic pulmonary fibrosis; SpO_2: peripheral capillary oxygen saturation

Correlation between the outcomes of 6MWT and cardiopulmonary function

SpO_2 was generally positively correlated to cardiopulmonary function, whereas the heart rate and Borg scale were negatively correlated. Specifically, the NYHA grade strongly correlated with 6MWD (r=0.82, p < 0.01). The 6MWD/predicted 6MWD had the highest correlation coefficient with FVC (r=0.30, p < 0.01) and FEV_1 (r=0.30, p < 0.01). SpO_2 at the end of 3 min had the strongest correlation to DL_{CO} (r=0.41, p < 0.01) (**Table 4).**

Table 4. Correlations between 6MWT outcomes and cardiopulmonary function.

For peer review only

**Outcomes of 6MWT**		**NYHA**	**MAP before 6MWT**	**MAP after 6MWT**	**MAP change**	**FVC**	$\mathbf{FEV_1}$	$\mathbf{DL_{CO}}$
6MWD (meter)	*r*	**0.82****	0.07*	0.09*	0.08*	0.24**	0.17**	0.26**
	n	751	949	733	733	846	846	806
6MWD / predicted 6MWD	*r*	0.64**	0.09**	0.14**	0.09*	**0.30****	**0.30****	0.28**
	n	751	949	733	733	846	846	806
SpO_2 at 0 min (%)	*r*	0.29**	0.03	0.01	-0.02	0.18**	0.17**	0.26**
	n	750	746	732	732	644	644	604
SpO_2 at end of 1 min (%)	*r*	0.27**	0.02	0.01	0.01	0.24**	0.21**	0.31**
	n	748	744	730	730	642	642	602
SpO_2 at end of 2 min (%)	*r*	0.28**	0.05	0.03	-0.01	0.27**	.23**	.37**
	n	749	745	731	731	643	643	603
SpO_2 at end of 3 min (%)	*r*	0.29**	0.047	0.034	0.00	**0.29****	**0.24****	**0.41****
	n	749	745	731	731	643	643	603
SpO_2 at end of 4 min (%)	*r*	0.27**	0.03	0.03	0.01	0.27**	0.23**	0.40**
	n	749	745	731	731	643	643	603
SpO_2 at end of 5 min (%)	*r*	0.27**	0.03	0.03	0.01	0.27**	0.23**	0.39**
	n	749	745	731	731	643	643	603
SpO_2 at end of 6 min (%)	*r*	0.26**	0.03	0.02	0.01	0.27**	0.22**	0.37**
	n	748	744	730	730	642	642	602

SpO_2 nadir (%)	*r*	0.27**	0.05	0.04	0.01	0.28**	0.24**	0.38**
	n	750	746	732	732	644	644	604
SpO_2 change (%)	*r*	-0.18**	-0.02	-0.02	-0.02	-0.24**	-0.19**	-0.32**
	n	748	744	730	730	642	642	602
Borg scale at 0 min	*r*	-0.23**	-0.02	0.02	0.05	-0.12**	-0.06	-0.13**
	n	727	926	714	714	829	829	791
Borg scale at the end	*r*	-0.25**	-0.05	0.00	0.07	-0.15**	-0.11**	-0.19**
	n	726	925	713	713	828	828	790
HR at 0 min (times/min)	*r*	-0.02	0.05	0.03	-0.01	-0.18**	-0.21**	-0.10*
	n	750	746	732	732	644	644	604
HR at end of 1 min (times/min)	*r*	0.16**	0.09**	0.106**	0.04	-0.08	-0.12**	0.00
	n	749	745	731	731	643	643	603
HR at end of 2 min (times/min)	*r*	0.18**	0.09*	0.13*	0.07	-0.08*	-0.11**	-0.02
	n	749	745	731	731	643	643	603
HR at end of 3 min (times/min)	*r*	0.18**	0.12**	0.16**	0.09*	-0.08	-0.11**	-0.01
	n	749	745	731	731	643	643	603
HR at end of 4 min (times/min)	*r*	0.19**	0.10**	0.14**	0.08*	-0.06	-0.11**	-0.03
	n	749	745	731	731	643	643	603
HR at end of 5 min (times/min)	*r*	0.22**	0.11**	0.16**	0.10**	-0.05	-0.09*	-0.02
	n	749	745	731	731	643	643	603
HR at end of 6 min (times/min)	*r*	0.24**	0.10**	0.16**	0.11**	-0.04	-0.09*	-0.02
	n	749	745	731	731	643	643	603

For peer review only

HR zenith (times/min)	*r*	0.19**	0.10**	0.12**	0.06	-0.03	-0.09*	0.01
	n	750	746	732	732	644	644	604
HR change (times/min)	*r*	-0.32**	-0.08*	-0.17*	-0.15**	-0.14**	-0.11**	-0.09*
	n	749	745	731	731	643	643	603

** means p < 0.01, * means p <0.05. 6MWT: six-minute walk test; 6MWD: six-minute walk distance; SpO2: peripheral capillary oxygen saturation; HR: heart rate; NYHA: New York Heart Association; MAP: mean arterial pressure; FVC: forced vital capacity; FEV_1: forced expiratory volume in the first second; DL_{CO}: diffusing capacity of the lungs for carbon monoxide.

For peer review only

Discussion

This study described identified the correlation between 6MWT outcomes and cardiopulmonary function and compared the difference between sub-types of ILD on 6MWT outcomes. We found that the HR and SpO_2 did not increase or decrease uniformly during walking. For approximately 10% of the patients, the SpO_2 nadir was lower than 80%, but they completed the test. Besides, SpO_2 nadir appeared at the end of the fourth minute for approximately 80% of patients. Therefore, 6MWD and SpO_2 had the strongest correlation with heart function and lung function of ILD, respectively. Moreover, group comparisons revealed that the 6MWD and SpO_2 change were more distinguishing for the subgroups of ILD.

Compared to previous studies, the average 6MWD in our study was 457.28 meters, which was moderate.[27,28] Heart rate increased continuously and SpO_2 decreased, with a slight rise in the fifth minute. However, the results of a previous study showed a slight increase in the fourth minute and a sharp drop in SpO_2.[29] Since our study had a bigger sample size, the average 6MWD and tendency of SpO_2 were more representative. According to the standard, 6MWT should be terminated when SpO_2 falls below 80%.[30] When SpO_2 was less than 88%, it was considered as a significant desaturation, and patients were recommended to take an oxygen supplement.[31,32,33] Without oxygen supplements in our study, SpO_2 nadir were lower than 88% and 80% for 30.1% and 9.3% of the patients, respectively. They all completed 6MWT without any chest pain, leg cramps, unsteady gait, diaphoresis, or a pale/ashen appearance, experiencing breathlessness, or reporting being too tired to continue,. Our

findings indicated that it is unwarranted to stop 6MWT when ILD patients only experience desaturation without other indications of termination, which corroborate the findings of Afzal et al.[34] The SpO_2 nadir is an essential outcome of 6MWT, and our research revealed that for 63.87% and 78.27% of the participants, SpO_2 nadir appeared at the end of the third and fourth minute respectively. Oxygen titration is generally performed with 6MWT to determine the oxygen flow that prevents oxygen saturation from falling below 88%, measured using pulse oximetry (SpO_2). According to Giovacchini et al.,[35] after a certain dose of oxygen is administered, the patients' SpO_2 should exceed 88% and be stable for three minutes. In our study, we found that the SpO_2 nadir for approximately 80% of the patients had appeared at the end of the fourth minute; hence, we strongly recommend that oxygen titration should be for four minutes.

Garin et al. did not find significant differences between IPF and SSc-ILD on 6MWD and dyspnea,[36] while Someya et al. found that patients with IPF had lower SpO_2 and higher Borg score than patients with dermatomyositis.[37] We observed no significant differences between the sub-types for Borg score and SpO_2 after walking. Since previous studies merely compared two different subgroups of ILD, our results were more comprehensive and reliable. Contrary to dyspnea and SpO_2 after walking, we found significant differences between groups on 6MWD and SpO_2 change. Therefore, 6MWD and SpO_2 change were the more distinguishing outcomes for sub-types of ILD. Although the HR at the 0 min, end of 6 min, and HR zenith showed significant differences between the sub-types, this finding was unclear because baseline HR showed differences before walking. Therefore, future studies in another population or multi-center may reinforce the findings.

Similar to previous studies, 6MWD and SpO_2 positively correlated with cardiopulmonary function outcomes such as NYHA, FVC, FEV_1, and DL_{CO},[5,8-10] while Borg score was negatively correlated.[30] Compared to 6MWD and SpO_2, the patients' heart rate had a weaker positive correlation to cardiopulmonary function outcomes. Hence, SpO_2 and distance were more valuable than heart rate in predicting the patients' cardiopulmonary function and degree of dyspnea. In a study, 6MWD was more correlated to DL_{CO} than SpO_2[27]; however, our result was to the contrary—lower FVC and DL_{CO} were associated with poor prognosis and high mortality.[16,38-40] Nevertheless, the DL_{CO} level was more valuable than FVC, as it captured the combined impact on the pulmonary reserve of IPF, emphysema, and pulmonary hypertension.[41] Since SpO_2 had the strongest correlation with DL_{CO}, we recommend

For peer review only

clinical practitioners to monitor the SpO_2 of ILD patients. Besides, the most valuable SpO_2 time point remains unclear. A previous study revealed that SpO_2 nadir and SpO_2 change had the same degree of correlation with DL_{CO},[42] and another study also highlighted the critical predictive value of the SpO_2 nadir.[43]In contrast, our findings illustrated that SpO_2 at the end of the third minute was more predictive than the SpO_2 nadir and SpO_2 change in DL_{CO}. Although 6MWD /predicted 6MWD had a higher correlation to FEV_1 and FVC than SpO_2 at the end of the third minute, 6MWD was more susceptible to factors such as age, sex, shorter corridor, and inappropriate walking shoes.[44-46] Furthermore, DL_{CO} was considered more critical than FEV_1 and FVC for ILD. Hence, the third-minute SpO_2 can be an alternative to predict lung function in ILD patients.

Limitations and future research directions

This study had several limitations. First, we did not conduct the second 6MWT for patients, and so the measured distance might not be the longest potential distance. Second, 25% of the values regarding some non-critical variables were missing, which might introduce selection bias and affect the validity and representativeness of the results. Replication in another population or a multicentric study could reinforce the findings. Third, lack of follow up on patients' prognosis and mortality hindered the prediction of 6MWT outcomes on the long-term clinical outcomes. Future research is required to explore the association between the outcomes of 6MWT and long-term prognosis.

Conclusions

Despite the above limitations, this study showed that increased HR and decreased SpO_2 during the 6MWT do not change uniformly. Approximately 10% of the patients, whose SpO_2 was less than 80%, completed 6MWT without any discomfort indicated. Hence, it is unwarranted to halt 6MWT when ILD patients experience only desaturation, without other indications of termination. SpO_2 nadir appeared at the end of the fourth minute for approximately 80% of the patients, which provides an important evidence regarding oxygen titration i.e., it is better to maintain SpO_2 above 88% for four minutes. Besides, the third-minute SpO_2 can be an alternative to predict patients' lung function. Conclusively, 6MWD and SpO_2 change showed significant differences between the sub-types of ILD, which indicated that they were more distinguishing for the sub-types of ILD.

Acknowledgments

For peer review only

The authors thank the other medical works for facilitating our data collection, they are Yanni XU, Yanfen PAN, Yanqiu LV and Xian LI. We would also like to thank Editage (www.editage.cn) for English language editing.

Authors' contributions

Jiaying LI and Xiaobing WU were involved in the design of the study. Miaozhen DENG, Xiaoyan LI, and Huiqun WEI were responsible for the data collection. Xinyin LIANG was involved in data analysis. Jiaying LI drafted the manuscript. All the authors made contributions to the revision and approved the final version.

Funding

This research was supported by the First Affiliated Hospital of Guangzhou Medical University, and the funding number is ZH201822.

Ethics approval

Ethical principles of the Declaration of Helsinki was utilized for guide this study, and the research protocol has been approved by the ethics committee of the First Affiliated Hospital of Guangzhou Medical University. The approval number is 2020 No.K-45.

Competing interests

The authors declare no competing interests.

Data sharing statement

Data were avaliable upon resonable request from Xiaobing Wu (wuxiaobing_gz@163.com).

Reference

1 Flaherty K R , Brown K K , Wells A U , et al. Design of the PF-ILD trial: a double-blind, randomised, placebo-controlled phase III trial of nintedanib in patients with progressive fibrosing interstitial lung disease. BMJ Open Respiratory Research, 2017, 4(1). https://doi.org/10.1136/bmjresp-2017-000212

2 Mikolasch, T. A., Porter, J. C. Transbronchial cryobiopsy in the diagnosis of interstitial lung disease:

For peer review only

a cool new approach. Respirology (Carlton, Vic.), 2014, 19(5), 623-624. https://doi.org/10.1111/resp.12320

3 Swigris, J. J., Stewart, A. L., Gould, M. K., et al. Patients' perspectives on how idiopathic pulmonary fibrosis affects the quality of their lives. Health and quality of life outcomes, 2005, 3(1), 61. https://doi.org/10.1186/1477-7525-3-61

4 ATS Committee on Proficiency Standards for Clinical Pulmonary Function Laboratories. ATS statement: guidelines for the six-minute walk test. Am J Respir Crit Care Med, 2002, 166, 111-117. https://doi.org/10.4067/S0717-73482009000100003

5 Caminati, A., Bianchi, A., Cassandro, R., et al. Walking distance on 6-MWT is a prognostic factor in idiopathic pulmonary fibrosis. Respiratory medicine, 2009, 103(1), 117-123. https://doi.org/10.1016/j.rmed.2008.07.022

6 Lancaster, L. H. Utility of the six-minute walk test in patients with idiopathic pulmonary fibrosis. Multidisciplinary respiratory medicine, 2018, 13(1), 1-7. https://doi.org/10.1186/s40248-018-0158-z

7 Wallaert, B., Monge, E., Le Rouzic, O., et al. Physical activity in daily life of patients with fibrotic idiopathic interstitial pneumonia. Chest, 2013, 144(5), 1652-1658. https://doi.org/10.1378/chest.13-0806

8 Bois, R. M. D., Weycker, D., Albera, C., et al. Six-minute-walk test in idiopathic pulmonary fibrosis: test validation and minimal clinically important difference. American Journal of Respiratory and Critical Care Medicine, 2011, 183(9), 1231-1237. https://doi.org/10.1164/rccm.201007-1179OC

9 Nathan, S. D., Du Bois, R. M., Albera, C., et al. Validation of test performance characteristics and minimal clinically important difference of the 6-minute walk test in patients with idiopathic pulmonary fibrosis. Respiratory medicine, 2015, 109(7), 914-922. https://doi.org/10.1016/j.rmed.2015.04.008

10 Mura, M., Ferretti, A., Ferro, O.,et al. Functional predictors of exertional dyspnea, 6-min walking distance and HRCT fibrosis score in idiopathic pulmonary fibrosis. Respiration, 2006, 73(4), 495-502. https://doi.org/10.1159/000089656

For peer review only

11 Kozu, R., Jenkins, S., Senjyu, H. Evaluation of activity limitation in patients with idiopathic pulmonary fibrosis grouped according to Medical Research Council dyspnea grade. Archives of physical medicine and rehabilitation, 2014, 95(5), 950-955. https://doi.org/10.1016/j.apmr.2014.01.016

12 Manali, E. D., Lyberopoulos, P., Triantafillidou, C., et al. MRC chronic Dyspnea Scale: Relationships with cardiopulmonary exercise testing and 6-minute walk test in idiopathic pulmonary fibrosis patients: a prospective study. BMC pulmonary medicine, 2010, 10(1), 32. https://doi.org/10.1186/1471-2466-10-32

13 Eaton, T., Young, P., Milne, D., et al. Six-minute walk, maximal exercise tests: reproducibility in fibrotic interstitial pneumonia. American journal of respiratory and critical care medicine, 2005, 171(10), 1150-1157. https://doi.org/10.1164/rccm.200405-578OC

14 Lama, V. N., Flaherty, K. R., Toews, G. B., et al. Prognostic value of desaturation during a 6-minute walk test in idiopathic interstitial pneumonia. American journal of respiratory and critical care medicine, 2003, 168(9), 1084-1090. https://doi.org/10.1164/rccm.200302-219OC

15 Przybyowski T , Tomalak W , Siergiejko Z , et al. Polish Respiratory Society guidelines for the methodology and interpretation of the 6 minute walk test (6MWT). Pneumonologia i alergologia polska, 2015, 83(4):283-297.doi:10.5603/PiAP.2015.0048

16 Brown, A. W., Nathan, S. D. The value and application of the 6-minute-walk test in idiopathic pulmonary fibrosis. Annals of the American Thoracic Society, 2018, 15(1), 3-10. https://doi.org/10.1513/AnnalsATS.201703-244FR

17 Holland, A. E., Spruit, M. A., Troosters, T., et al. An official European Respiratory Society/American Thoracic Society technical standard: field walking tests in chronic respiratory disease. Eur Respir J, 2014, 44(6), 1428-46. https://doi.org/doi:10.1183/09031936.00150314

18 Miller-Davis, C., Marden, S., Leidy, N. K. The New York Heart Association Classes and functional status: what are we really measuring?. Heart & Lung, 2006, 35(4), 217-224. https://doi.org/10.1016/j.hrtlng.2006.01.003

19 Harvey, Réjane M. Nomenclature and Criteria for Diagnosis of Diseases of the Heart and Blood

For peer review only

Vessels. Journal of the American Medical Association, 1994, 153(9):2054-2054.

20 Borg G A, Psychophysical bases of perceived exertion .Med Sci Sports Exerc, 1982, 14: 377-81.https://doi.org/10.1249/00005768-198205000-00012

21 Williams, N. The Borg rating of perceived exertion (RPE) scale. Occupational Medicine, 2017, 67(5), 404-405. https://doi.org/10.1093/occmed/kqx063

22 Enright, P. L.. The six-minute walk test. Respiratory care, 2003, 48(8), 783-785. https://doi.org/10.1093/eurheartj/ehi452

23 Enright, P. L., & Sherrill, D. L. (1998). Reference equations for the six-minute walk in healthy adults. American journal of respiratory and critical care medicine, 158(5 Pt 1), 1384–1387. https://doi.org/10.1164/ajrccm.158.5.9710086

24 Spencer, L. M., Alison, J. A., & McKeough, Z. J. (2008). Six-minute walk test as an outcome measure: are two six-minute walk tests necessary immediately after pulmonary rehabilitation and at three-month follow-up?. American journal of physical medicine & rehabilitation, 87(3), 224–228. https://doi.org/10.1097/PHM.0b013e3181583e66

25 Hubbard, R., Johnston, I., Britton, J. Survival in patients with cryptogenic fibrosing alveolitis: a population-based cohort study. Chest, 1998, 113(2), 396-400. https://doi.org/10.1378/chest.113.2.396

26 Cottin, V., Hirani, N. A., Hotchkin, D. L.,et al. Presentation, diagnosis and clinical course of the spectrum of progressive-fibrosing interstitial lung diseases. European Respiratory Review, 2018, 27(150). https://doi.org/10.1183/16000617.0076-2018

27 Nishiyama, O., Yamazaki, R., Sano, H., et al. Pulmonary hemodynamics and six-minute walk test outcomes in patients with interstitial lung disease. Canadian respiratory journal, 2016. https://doi.org/10.1155/2016/3837182

28 Chetta, A., Aiello, M., Foresi, A., et al. Relationship between outcome measures of six-minute walk test and baseline lung function in patients with interstitial lung disease. Sarcoidosis, Vasculitis, and Diffuse Lung Diseases: Official Journal of WASOG, 2001, 18(2), 170-175.

For peer review only

https://doi.org/10.1053/rmed.2001.1087

29 Plessis J , Fernandes S , Jamal R , et al. Exertional hypoxemia is more severe in fibrotic interstitial lung disease than in COPD[J]. Respirology, 2017.

30 Holland AE, Spruit MA, Troosters T, et al. An offificial European Respiratory Society/American Thoracic Society technical standard: field walking tests in chronic respiratory disease. Eur Respir J 2014; 44:1428-46

31 Casanova C, Cote C, Marin JM et al. Distance and oxygen desaturation during the 6-min walk test as predictors of long-term mortality in patients with COPD. Chest 2008; 134: 746−752. doi: 10.1378/chest.08-0520.

32 Salzman SH. The 6-min walk test: clinical and research role, technique, coding, and reimbursement. Chest 2009;135(5): 1345e52.

33 Hook JL, Arcasoy SM, Zemmel D, Bartels MN, Kawut SM, Lederer DJ. Titrated oxygen requirement and prognostication in idiopathic pulmonary fifibrosis. Eur Respir J 2012;39(2): 359e65.

34 Afzal S , Burge A T , Lee A L , et al. Should the 6-Minute Walk Test Be Stopped If Oxyhemoglobin Saturation Falls Below 80%?[J]. Archives of Physical Medicine & Rehabilitation, 2018.

35 Giovacchini CX, Mathews AM, Lawlor BR, MacIntyre NR. Titrating Oxygen Requirements During Exercise: Evaluation of a Standardized Single Walk Test Protocol. Chest. 2018 Apr;153(4):922-928. doi: 10.1016/j.chest.2017.11.009.

36 Garin MC, Highland KB, Silver RM, Strange C. Limitations to the 6-minute walk test in interstitial lung disease and pulmonary hypertension in scleroderma. J Rheumatol. 2009 Feb;36(2):330-6. doi: 10.3899/jrheum.080447. Epub 2009 Jan 22.

37 Someya F, Mugii N. Limitations to the 6-minute walk test in dermatomyositis with interstitial lung disease in comparison with idiopathic interstitial pneumonia. Clin Med Insights Circ Respir Pulm Med. 2013;7:1-6. doi: 10.4137/CCRPM.S10764.

For peer review only

38 Holland, A. E., Dowman, L., Fiore, J.,et al. Cardiorespiratory responses to 6-minute walk test in interstitial lung disease: not always a submaximal test. BMC pulmonary medicine, 2014, 14(1), 136. https://doi.org/10.1186/1471-2466-14-136

39 Ostoji P , Damjanov N . Different clinical features in patients with limited and diffuse cutaneous systemic sclerosis. Clinical Rheumatology, 2006, 25(4):453-457. https://doi.org/10.1007/s10067-005-0041-0

40. Khanna Dinesh,Tashkin Donald P,Denton Christopher P et al. Etiology, Risk Factors, and Biomarkers in Systemic Sclerosis with Interstitial Lung Disease. Am J Respir Crit Care Med, 2020, 201: 650-660. https://doi.org/10.1164/rccm.201903-0563CI

41 Wells A U . Novel exploratory data in interstitial lung disease[J]. Respirology, 2019, 24(8).

42 Du Plessis JP, Fernandes S, Jamal R, Camp P, Johannson K, Schaeffer M, Wilcox PG, Guenette JA, Ryerson CJ. Exertional hypoxemia is more severe in fibrotic interstitial lung disease than in COPD. Respirology. 2018 Apr;23(4):392-398. doi: 10.1111/resp.13226.

43 Lettieri, C. J. , Nathan, S. D. , Browning, R. F. ,et al. The distance-saturation product predicts mortality in idiopathic pulmonary fibrosis. Respiratory Medicine, 2006, 100(10), 1734-1741. https://doi.org/10.1016/j.rmed.2006.02.004

44 Nishiyama O, Yamazaki R, Sano H, Iwanaga T, Higashimoto Y, Kume H, Tohda Y. Pulmonary Hemodynamics and Six-Minute Walk Test Outcomes in Patients with Interstitial Lung Disease. Can Respir J. 2016;2016:3837182. doi: 10.1155/2016/3837182. Epub 2016 May 18. PMID: 27445533; PMCID: PMC4904526.

45 Beekman E, Mesters I, Hendriks E.J et al. Course length of 30 metres versus 10 metres has a significant influence on Six-minute walk distance in patients with COPD: an experimental crossover study. J Physiother 2013; 59: 169−176. doi: 10.1016/S1836-9553(13)70181-4.

46 Barrios JA, Crenshaw JR, Royer TD, Davis IS. Walking shoes and laterally wedged orthoses in the clinical management of medial tibiofemoral osteoarthritis: a one-year prospective controlled trial. Knee 2009; 16: 136-42. doi: 10.1016/j. knee.2008.10.002.

For peer review only

Figure 1. Description of SpO_2 and HR in patients with ILD during 6MWT

Figure 2. Statistical analysis chart of SpO_2 in ILD patients within six minutes

For peer review only

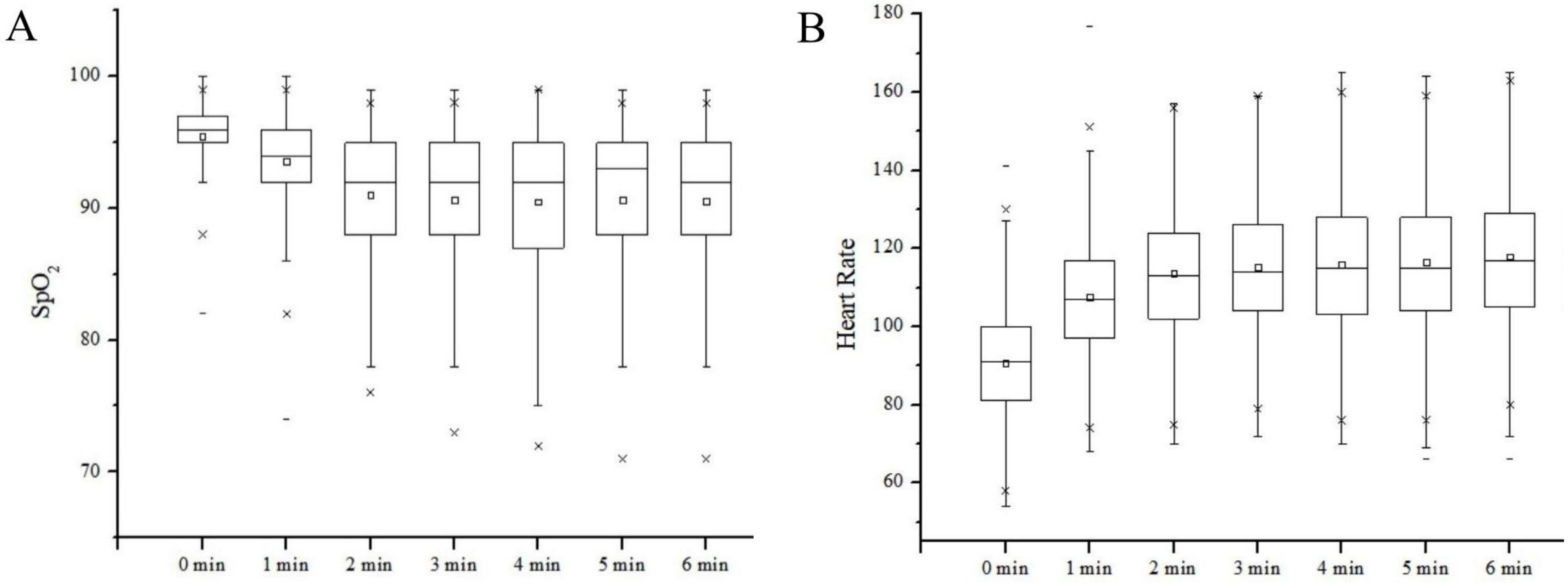
A
SpO2
100
90
80
70
0 min
1 min
2 min
3 min
4 min
5 min
6 min
B
Heart Rate
180
160
140
120
100
80
60
0 min
1 min
2 min
3 min
4 min
5 min
6 min

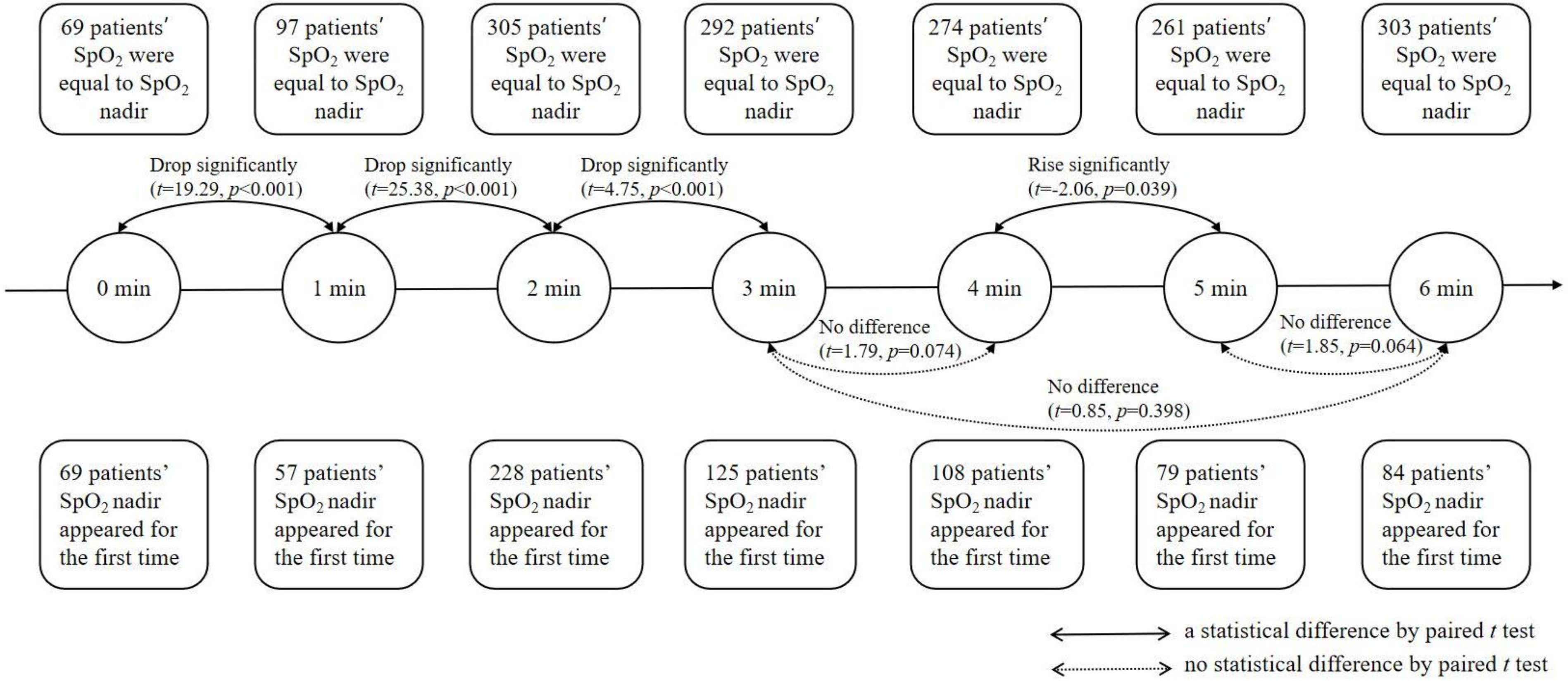
69 patients' SpO2 were equal to SpO2 nadir
97 patients' SpO2 were equal to SpO2 nadir
305 patients' SpO2 were equal to SpO2 nadir
292 patients' SpO2 were equal to SpO2 nadir
274 patients' SpO2 were equal to SpO2 nadir
261 patients' SpO2 were equal to SpO2 nadir
303 patients' SpO2 were equal to SpO2 nadir
Drop significantly (t=19.29, p<0.001)
Drop significantly (t=25.38, p<0.001)
Drop significantly (t=4.75, p<0.001)
Rise significantly (t=-2.06, p=0.039)
0 min
1 min
2 min
3 min
4 min
5 min
6 min
No difference (t=1.79, p=0.074)
No difference (t=1.85, p=0.064)
No difference (t=0.85, p=0.398)
69 patients' SpO2 nadir appeared for the first time
57 patients' SpO2 nadir appeared for the first time
228 patients' SpO2 nadir appeared for the first time
125 patients' SpO2 nadir appeared for the first time
108 patients' SpO2 nadir appeared for the first time
79 patients' SpO2 nadir appeared for the first time
84 patients' SpO2 nadir appeared for the first time
a statistical difference by paired t test
no statistical difference by paired t test

STROBE Statement—checklist of items that should be included in reports of observational studies

	Item No	Recommendation	Page No
**Title and abstract**	1	(*a*) Indicate the study's design with a commonly used term in the title or the abstract	1
		(*b*) Provide in the abstract an informative and balanced summary of what was done and what was found	1
**Introduction**			
Background/rationale	2	Explain the scientific background and rationale for the investigation being reported	1-2
Objectives	3	State specific objectives, including any prespecified hypotheses	2
**Methods**			
Study design	4	Present key elements of study design early in the paper	3
Setting	5	Describe the setting, locations, and relevant dates, including periods of recruitment, exposure, follow-up, and data collection	3
Participants	6	(*a*) *Cohort study*—Give the eligibility criteria, and the sources and methods of selection of participants. Describe methods of follow-up<br>*Case-control study*—Give the eligibility criteria, and the sources and methods of case ascertainment and control selection. Give the rationale for the choice of cases and controls<br>*Cross-sectional study*—Give the eligibility criteria, and the sources and methods of selection of participants	3
		(*b*) *Cohort study*—For matched studies, give matching criteria and number of exposed and unexposed<br>*Case-control study*—For matched studies, give matching criteria and the number of controls per case	-
Variables	7	Clearly define all outcomes, exposures, predictors, potential confounders, and effect modifiers. Give diagnostic criteria, if applicable	3-4
Data sources/ measurement	8*	For each variable of interest, give sources of data and details of methods of assessment (measurement). Describe comparability of assessment methods if there is more than one group	*4*
Bias	9	Describe any efforts to address potential sources of bias	4
Study size	10	Explain how the study size was arrived at	3
Quantitative variables	11	Explain how quantitative variables were handled in the analyses. If applicable, describe which groupings were chosen and why	4
Statistical methods	12	(*a*) Describe all statistical methods, including those used to control for confounding	4
		(*b*) Describe any methods used to examine subgroups and interactions	4
		(*c*) Explain how missing data were addressed	4
		(*d*) *Cohort study*—If applicable, explain how loss to follow-up was addressed<br>*Case-control study*—If applicable, explain how matching of cases and controls was addressed<br>*Cross-sectional study*—If applicable, describe analytical methods taking account of sampling strategy	-
		(*e*) Describe any sensitivity analyses	4

Continued on next page

For peer review only - http://bmjopen.bmj.com/site/about/guidelines.xhtml

**Results**			
Participants	13*	(a) Report numbers of individuals at each stage of study—eg numbers potentially eligible, examined for eligibility, confirmed eligible, included in the study, completing follow-up, and analysed	5
		(b) Give reasons for non-participation at each stage	5
		(c) Consider use of a flow diagram	5
Descriptive data	14*	(a) Give characteristics of study participants (eg demographic, clinical, social) and information on exposures and potential confounders	5
		(b) Indicate number of participants with missing data for each variable of interest	5
		(c) *Cohort study*—Summarise follow-up time (eg, average and total amount)	5
Outcome data	15*	*Cohort study*—Report numbers of outcome events or summary measures over time	-
		Case-control study—Report numbers in each exposure category, or summary measures of exposure	-
		Cross-sectional study—Report numbers of outcome events or summary measures	*5*
Main results	16	(*a*) Give unadjusted estimates and, if applicable, confounder-adjusted estimates and their precision (eg, 95% confidence interval). Make clear which confounders were adjusted for and why they were included	5
		(*b*) Report category boundaries when continuous variables were categorized	5
		(*c*) If relevant, consider translating estimates of relative risk into absolute risk for a meaningful time period	-
Other analyses	17	Report other analyses done—eg analyses of subgroups and interactions, and sensitivity analyses	5
**Discussion**			
Key results	18	Summarise key results with reference to study objectives	6-7
Limitations	19	Discuss limitations of the study, taking into account sources of potential bias or imprecision. Discuss both direction and magnitude of any potential bias	6-7
Interpretation	20	Give a cautious overall interpretation of results considering objectives, limitations, multiplicity of analyses, results from similar studies, and other relevant evidence	6-7
Generalisability	21	Discuss the generalisability (external validity) of the study results	6-7
**Other information**			
Funding	22	Give the source of funding and the role of the funders for the present study and, if applicable, for the original study on which the present article is based	8

*Give information separately for cases and controls in case-control studies and, if applicable, for exposed and unexposed groups in cohort and cross-sectional studies.

Note: An Explanation and Elaboration article discusses each checklist item and gives methodological background and published examples of transparent reporting. The STROBE checklist is best used in conjunction with this article (freely available on the Web sites of PLoS Medicine at http://www.plosmedicine.org/, Annals of Internal Medicine at http://www.annals.org/, and Epidemiology at http://www.epidem.com/). Information on the STROBE Initiative is available at www.strobe-statement.org.

For peer review only
